# A systematic review and bibliometric analysis of robot vs. laparoscopic surgery in urogynecology: current trends and future directions

**DOI:** 10.1007/s11701-025-02885-2

**Published:** 2025-11-03

**Authors:** Esra Bilir, Xezal Derin, Jasmina Veta Darkovski, Manou Manpreet Kaur, Johannes Ackermann, Nicolai Maass, Leila Allahqoli, Rufus Cartwright, Ibrahim Alkatout

**Affiliations:** 1 Department of Gynecologic Oncology, Koç University School of Medicine, Istanbul, Turkey; 2 Department of Obstetrics and Gynecology, University Hospitals Schleswig-Holstein, Campus Kiel, Arnold-Heller-Str. 3, Haus C, 24105 Kiel, Germany; 3 Department of Gynecology and Tumor Surgery, Charite Comprehensive Cancer Center, Berlin, Germany; 4 Division Obstetrics and Prenatal Medicine, Goethe University Frankfurt-Main, Frankfurt, Germany; 5 Department of Gynaecology and Obstetrics, University Hospital Ulm, Ulm, Germany; 6 Chelsea and Westminster Hospital NHS Foundation Trust, London, UK; 7 Ministry of Health and Medical Education, Tehran, Iran; 8 Department of Gynecology and Obstetrics, University Medical Center Göttingen, Göttingen, Germany

**Keywords:** Robot-assisted surgery, Urogynecology, Laparoscopy, Minimally invasive surgery, Future, Sacrocolpopexy

## Abstract

We aim to evaluate the current role of robot-assisted surgery in urogynecology by comparing its outcomes and applications to those of laparoscopic surgery. We ran a computed search on PubMed, Cochrane Library, Scopus, Web of Science, and Ovid MEDLINE in January 2024. Our review followed the Preferred Reporting Items for Systematic Reviews and Meta-Analyses guidelines and was registered in PROSPERO (CRD42024500936). Our search strategy targeted commonly performed urogynecological surgeries, such as Burch colposuspension, fistula repair, urethropexy, pelvic organ prolapse (POP), sacrohysteropexy, sacrocolpopexy, cervicosacropexy, enterocele, cystocele, urinary incontinence, pectopexy, cervicopectopexy, colpopectopexy, native tissue repair, and anterior and posterior colporrhaphy. We included only original studies comparing robot-assisted and laparoscopic approaches in urogynecological surgeries. Additionally, we conducted a bibliometric analysis of the selected studies. Our final analysis included 36 studies, where 32 studies focused on POP, with sacrocolpopexy being the most frequently performed procedure (88.9%). Most of the studies originated from high-income countries. Most studies were retrospective (63.9%). The analysis included data on 29,172 patients, with 52.5% undergoing laparoscopy and 47.5% undergoing robot-assisted surgery. While no significant differences were observed in complications such as mesh erosion, the robot-assisted surgery group exhibited a higher incidence, where all of them were sacrocolpopexy cases. The follow-up duration ranged from one week to three years, with substantial variability in the reporting of surgical duration and complications. Although robot-assisted surgery might promise improvements in urogynecology, particularly in sacrocolpopexy, the comparison with laparoscopy is inconclusive in terms of patient outcomes and complications. Further standardization of reporting and long-term data are needed to refine these findings. The major limitation of our study was the high heterogeneity among the studies.

**Supplementary Information:**

The online version contains supplementary material available at 10.1007/s11701-025-02885-2.

## Introduction

Robot-assisted surgery, as a prominent and progressive minimally invasive technique, has gained increasing traction within the field of gynecology. Its utilization has become particularly widespread in benign gynecological procedures, such as hysterectomy, endometriosis surgery, and myomectomy [1]. Robot-assisted surgery represents the most advanced approach to minimally invasive procedures today [2]. The integration of 3D technology enhances surgical field visualization, while the extension of surgical instruments to seven degrees of freedom enables precise maneuverability, making minimally invasive techniques feasible even in complex cases [2].

In contrast, traditional laparoscopy has still been accepted as the standard for many gynecological surgeries, including those in the subspecialty of urogynecology [3]. Many urogynecological procedures, such as vaginal prolapse repair, were historically performed by open surgery, vaginally or via conventional laparoscopy. However, as demographic shifts, including aging populations and rising obesity rates, increase the demand for urogynecological interventions, there is growing interest in the optimal surgical access. By 2050, it is estimated that approximately one-third of the adult female population in the United States will experience at least one pelvic floor disorder [4].

The debate on vaginal meshes intensified after a 2016 Cochrane meta-analysis highlighted the adverse effects associated with vaginal alloplastic mesh operations, leading to bans on vaginal mesh use in countries such as the United States, the United Kingdom, and Australia [5, 6]. These restrictions have necessitated a reliance on minimally invasive surgeries for mesh-based prolapse surgeries in many settings, while other nations have imposed complete bans on mesh utilization.

In a meta-analysis comparing robot-assisted sacrocolpopexy and laparoscopic sacrocolpopexy involving 1157 patients, the robot-assisted approach was associated with increased postoperative pain and longer operating times [7]. Nevertheless, the authors reported no significant differences in anatomical outcomes, mortality, hospital stay, and postoperative quality of life [7]. Another meta-analysis on robot-assisted sacrocolpopexy concluded that it is an effective surgical approach for treating apical prolapse, demonstrating a high anatomic cure rate and a low incidence of complications [8].

The in-patient data from the German Federal Statistical Office, covering the period from 2006 to 2021, included a total of 1,150,811 surgical procedures [9]. The authors observed that while the trends in transvaginal mesh surgery for the anterior compartment remained relatively stable (*p* = 0.147), a significant decline was noted in the other compartments, including the posterior (*p* < 0.001) and enterocele surgeries (*p* < 0.001) [9].

Despite the growing adoption of robot-assisted surgery, there is still a lack of comprehensive comparisons between robot-assisted and laparoscopic approaches across various urogynecological procedures. The primary objective of our systematic review is to analyze the existing literature to evaluate the current evidence comparing robot-assisted and laparoscopic surgery in the field of urogynecology. As a secondary objective of our study, we conducted an independent evaluation of research comparing laparoscopy with robot-assisted approaches. Additionally, we conducted a bibliometric analysis of the selected studies.

## Materials and methods

In our review, we used two distinct methodological approaches: first, a systematic review to evaluate comparative studies on robot-assisted versus laparoscopic surgery in urogynecology, and second, a bibliometric analysis of the included studies to assess publication patterns, journal distribution, and citation metrics.

### Search strategy

We conducted a systematic literature review using a comprehensive electronic search strategy. The search was performed across multiple databases, including PubMed, Cochrane Library, Scopus, Web of Science, and Ovid MEDLINE in January 2024. Our query consisted of three distinct components. The first component focused on keywords related to robot-assisted surgery, the second encompassed keywords pertaining to laparoscopy, and the third targeted terms associated with urogynecologic surgeries. The surgical terms included in the search were Burch, colposuspension, fistula, urethropexy, pelvic organ prolapse, prolapse, sacrohysteropexy, sacrocolpopexy, cervicosacropexy, CSP, sacral colpopexy, enterocele, cystocele, incontinence, incontinent, mesh, band, type, repair, pectopexy, cervicopectopexy, colpopectopexy, native tissue repair, anterior colporrhaphy, anterior vaginal wall repair, posterior colporrhaphy, and posterior vaginal wall repair. These terms were meticulously selected and reviewed by the senior authors of the project to ensure their relevance and comprehensiveness. We incorporated all relevant Medical Subject Headings (MeSH) related to robot-assisted surgery, laparoscopy, and the specified urogynecological procedures. Additionally, we supplemented these with manually curated non-MeSH keywords to ensure comprehensive coverage across the various databases. The complete search strategy is available in Supplementary Information 1. The electronic search was conducted by an experienced medical librarian from Koç University Health Sciences Library, Istanbul, Türkiye. Furthermore, we prospectively registered our study protocol in PROSPERO (registration number CRD42024500936) on 25 January 2024 [10].

### Selection of studies

We included only original research articles, excluding case reports, congress abstracts, editorials, commentaries, book chapters, ongoing clinical trials, study protocols, retracted papers, and all types of review articles. Studies were eligible if they included only female participants; those involving males were excluded unless a separate analysis for females was performed. We included the patients with the same primary diagnosis and the same primary surgery. Thus, the studies comparing different conditions or surgical techniques between laparoscopy and robot-assisted approaches were excluded. Furthermore, we excluded the studies on vaginal natural orifice transluminal endoscopic surgery, mini-laparoscopy, and single-port surgeries.

Eligible studies were required to have full-text availability in English and to explicitly compare outcomes of a specific urogynecological surgery performed via laparoscopy versus a robot-assisted approach. Study selection and data extraction were conducted using Covidence software (Veritas Health Innovation Ltd., Melbourne, Australia) [11]. Four co-authors (E.B., X.D., J.V., M.M.K.) independently screened studies for eligibility. Discrepancies were resolved by the primary investigator (E.B.) after discussion with another member of the screening team. The selection process adhered to the Preferred Reporting Items for Systematic Reviews and Meta-Analyses (PRISMA) 2020 guidelines [12]. Covidence facilitated a structured workflow, beginning with the screening of titles and abstracts and progressing to full-text reviews [11]. During this process, we manually removed the further duplicates. Following the finalization of full texts, EndNote® 20.6 for Mac OS software was used for reference management, culminating in data extraction from the final eligible studies.

### Data extraction

Upon finalizing the study selection process, the following data were extracted from the full-text articles: author, year of publication, country of the study, study design, urogynecological condition, intervention, information on mesh, total number of patients, number of patients undergoing laparoscopy, number of patients undergoing robot-assisted surgery, robot surgical system, baseline characteristics for comparison, intraoperative variables for comparison, and postoperative outcomes for comparison. We classified the countries into three income groups: low-middle-income (LMIC), middle-income (MIC), and high-income (HIC) based on the World Health Organization’s (WHO) database [13].

We also assessed whether the studies reported outcomes in accordance with the recommendations outlined in the National Institutes of Health Terminology Workshop for Researchers in Female Pelvic Floor Disorders, which was published in 2001 [14]. Specifically, we examined the references to determine if the original recommendations were appropriately cited and carefully reviewed the reported outcomes to verify adherence to these guidelines.

### Bibliometric analysis

We accessed the websites of the journals to determine their impact factors. Additionally, we created a word cloud analysis illustration based on the terms used in the titles of the studies. We retrieved both the total and yearly citation counts for each article from Google Scholar on October 4, 2025. [15]

### Statistics

Frequencies, summaries, and percentages were calculated if necessary. Moreover, we pooled the patient data when needed. We used Python 3.0 for the calculations. The analysis was performed using Python 3.0 scripts executed on Google Colab, an interactive computing environment [16]. Our codes are available in Supplementary Information 2. We accepted a p-value of less than 0.05 as the threshold for statistical significance in all tests.

## Results

**Fig. 1:**
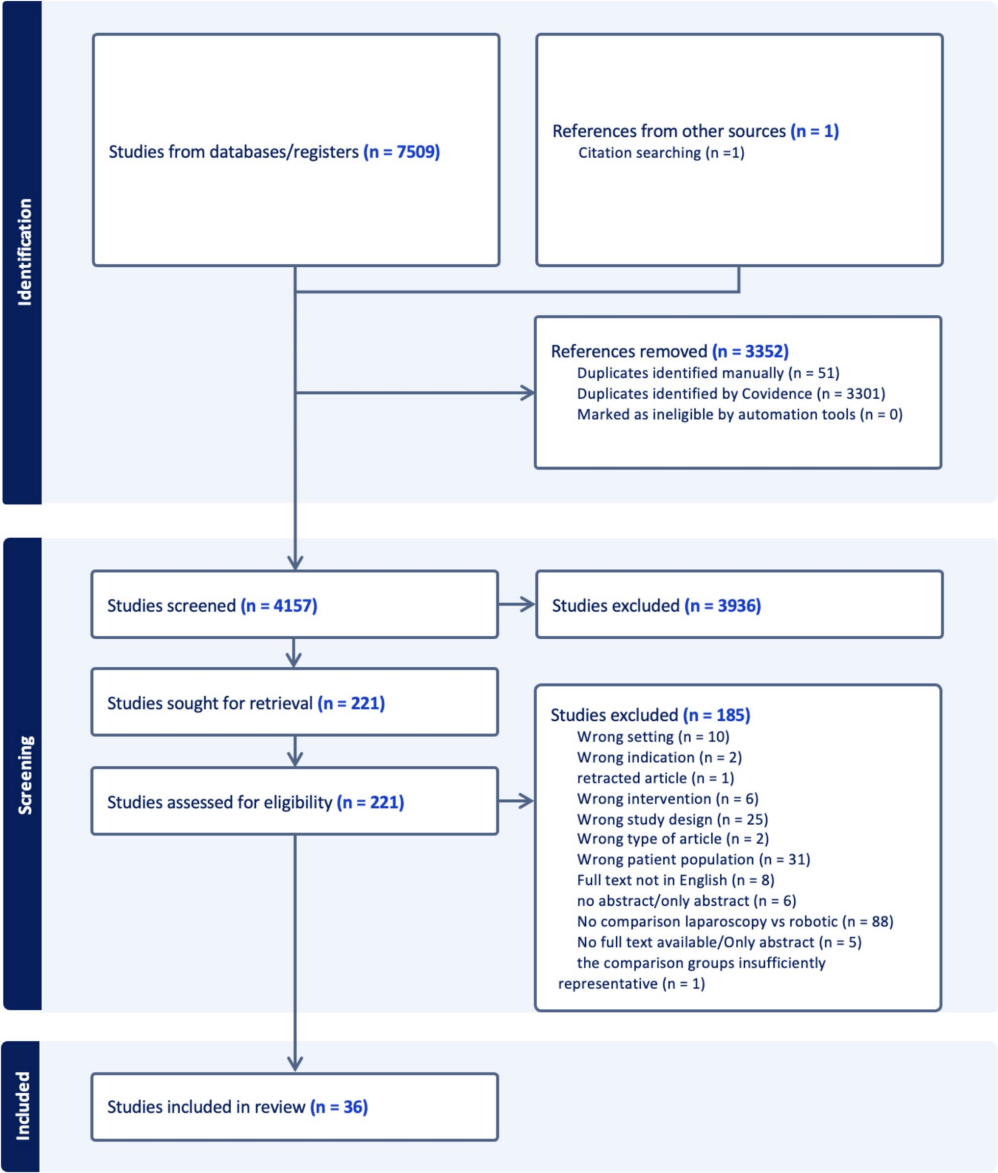
PRISMA flowchart: a systematic overview of the study selection process, illustrating the number of records identified, screened, excluded, and included in the final analysis according to PRISMA guidelines

**Fig. 2:**
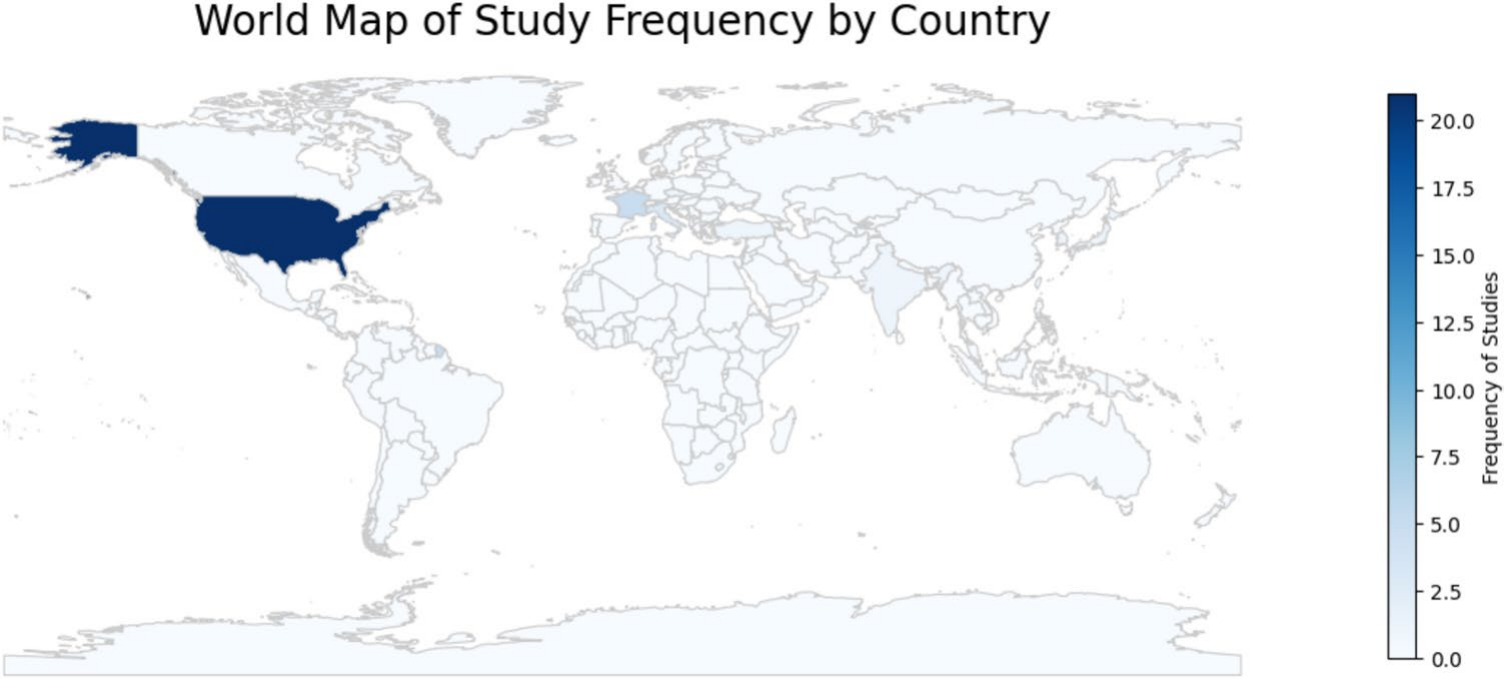
Country heatmap: geographical distribution of the included studies, highlighting the frequency of contributions from different countries

**Fig. 3:**
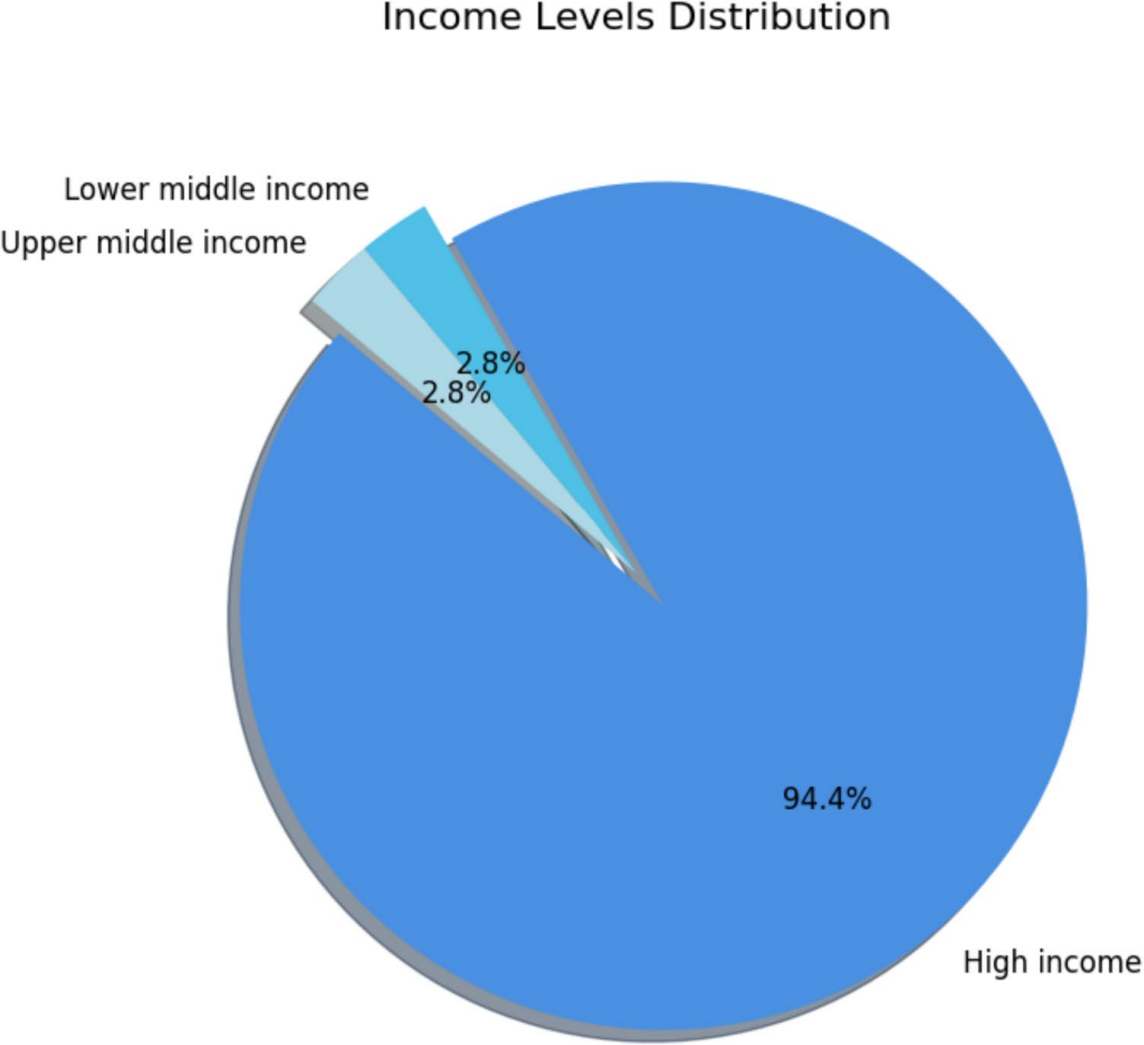
Country income level distribution: a pie chart showing the distribution of included studies across countries categorized by WHO income levels (low, middle, and high income)

A total of 7509 studies were identified through our search strategy across the specified databases. Following the removal of duplicates, 4137 studies remained for screening. The detailed selection process is illustrated in the PRISMA flowchart (Fig. 1). The baseline characteristics of the 36 included studies, published between 2009 and 2023, are summarized in Table 1. The distribution of countries is illustrated in Fig. 2, with the majority originating from high-income countries (HICs) (94.4%) (Fig. 3).

**Table 1:** Baseline characteristics of the included studies

Author, year	Country	Study design/center	Total patients	Urogynecological condition (n/total, %)	Intervention, mesh, concomitant surgeries	Outcome	Laparoscopy (N/Total, %)	Robot (N/Total, %)	Robot surgical system
1. Patel et al. 2009 [26]	USA	Retrospective NR	10	Prolapse stage 3 (10/10, 100%)	Sacrocolpopexy Concomitant surgeries - Paravaginal defect repair or Burch - Posterior colporrhaphy - Cystourethroscopy	Cost (in US Dollars)	5/10 (50.0%)	5/10 (50.0%)	NR
2. White et al. 2009 [27]	USA	Retrospective Single center	20	POP - Symptomatic Stage 2 (5/20, 25.0%) - Stage 3 (14/20, 70.0%) - Stage 4 (1/20, 5.0%)	Sacrocolpopexy polypropylene mesh (1.1 × 30 cm) Concomitant surgeries - TVT: 3 in LS and 2 in RS - TOT: 3 in LS and 3 in RS - Vaginal Procedure Cystocele: 2 in LS and 2 in RS - Vaginal Procedure Rectocele: 1 in RS	Evaluate short- term outcomes after SCP	10/20 (50.0%)	10/20 (50.0%)	NR
3. Judd et al. 2010 [28]	USA	Modeling Single center	NR	Prolapse	Sacrocolpopexy synthetic polypropylene mesh	Cost-Minimization analysis (in US Dollars)	NR	NR	daVinci HD S Robot Surgical System (Intuitive Surgical, Inc.)
4. Chan et al. 2011 [29]	Hong Kong	Retrospective Single center	36	Vaginal vault prolapse	Sacrocolpopexy Y-shaped polypropylene mesh (Ethicon, Inc, Somerville, NJ) Concomitant surgeries - Pelvic floor repair surgery: 15 in LS, 15 in RS - Colposuspension: 4 in LS and 1 in RS - TVT: 4 in LS and 2 in RS	medium-term outcome	20/36 (55.6%)	16/36 (44.4%)	2007–2008: a three-arm robot (da Vinci Surgical System; Intuitive Surgical, Sunnyvale [CA], US) when became available: a four-arm robot (da Vinci Surgical System, Intuitive Surgical)
5. Paraiso et al. 2011 [30]	USA	Blinded randomized trial Single center	Planned: 78 Final: 68	Stage 2– 4 posthysterectomy vaginal prolapse	Sacrocolpopexy two separate 4 × 15-cm pieces of poly- propylene mesh Concomitant surgeries - incontinence surgery: 23 in LS and 25 in RS - Rectocele repair: 16 in LS and 10 in RS	LS vs RS in Sacrocolpopexy	38/78 (48.7%) 33/68 (48.5%)	40/78 (51.3%) 35/68 (51.5%)	da Vinci Surgical System
6. Tan-Kim et al. 2011 [31]	USA	Retrospective Multicenter	104	Vaginal vault prolapse	Sacrocolpopexy polypropylene mesh Concomitant surgeries - Mid-urethral sling: 29 in LS and 9 in RS - Posterior repair: 6 in LS and 3 in RS	LS vs RS in Sacrocolpopexy	61/104 (58.7%)	43/104 (41.3%)	da Vinci robot-S (Intuitive Surgical Inc., Sunnyvale, Calif)
7. Wong et al. 2011 [32]	France	Prospective Nonrandom Single center	63	Complex rectocele	Rectopexy either a single-mesh fixation for posterior-compartment prolapse (rectoenterocele) or double-mesh fixation for combined anterior-compartment (with cystocele) and posterior-compartment prolapse Concomitant surgeries - TVT: 6 in LS and 3 in RS (P = 0 > 0.999)	LS vs RS	40/63 (63.5%)	23/63 (36.5%)	4-armed Da Vinci-S surgical system (Intuitive Surgical Inc., Sunnyvale, CA)
8. Antosh et al. 2012 [33]	USA	Retrospective Single center	88	Stage II/IV vaginal prolapse	Sacrocolpopexy - Synthetic polypropylene mesh was used in all cases (Gynecare Gynemesh [Ethicon, Somerville, NJ], Restorelle mesh [Mpathy Medical, Raynham, Mass], or IntePro mesh [American Medical Systems, Minnetonka, Minn]) Concomitant surgeries - Hysterectomy: 9 in LS and 28 in RS (*P* = 0.74) - Sling: 11 in LS and 20 in RS (*P* = 0.14) - posterior repair: 18 in LS and 32 in RS (*P* = 0.016)	LS vs RS in Sacrocolpopexy operative times and short-term outcome	23/88 (26.1%)	65/88 (73.9%)	da Vinci S system (Intuitive Surgical) and 5 operative ports
9. Pulliam et al. 2012 [34]	USA	Retrospective Single center	91	Vaginal vault prolapse or advanced uterine prolapse	Apical Sacropexy (Sacrocolpopexy) with Y-shaped piece of polypro- pylene mesh (tailored out of Gynecare Gynemesh PS, (Ethicon, Somerville, NJ) Concomitant surgeries - Hysterectomy: 10 in LS and 4 in RS (*P* = 0.7) - Suburethral sling: 16 in LS and 15 in RS (*P* = 0.9) - Mesh excision: 5 in LS and 1 in RS (*P* = 0.1) - Colporrhaphy: 9 in LS and 5 in RS (*P* = 1) - Oophorectomy: 6 in LS and 6 in RS (*P* = 0.8) - Lysis of adhesions: 13 in LS and 10 in RS (*P* = 0.7) - Hysteropexy: 1 in LS and 2 in RS (*P* = 0.5)	- Primary outcome: operative time - Secondary out- comes: EBL, rate of conversion, intraoperative complications, hospital stay, and objective prolapse outcome - Robot learning curve	48/91 (52.7%)*	43/91 (47.3%)*	NR
10. Seror et al. 2012 [35]	France	Prospective Single center	67	POP	Sacrocolpopexy - Y-shaped polypropylene mesh (Parie- tex®, Tyco Healthcare, Gosport, UK) Concomitant surgeries - TVT: 24 in LS and 3 in RS (*P* = 0.006) - TOT: 1 in LS and 3 in RS (*P* = 0.006)	Short-term functional outcomes	47/67 (70.1%)	20/67 (29.9%)	NR
11. Awad et al. 2013 [36]	Israel	Retrospective Single center	80	Vaginal apex prolapse	Sacrocolpopexy - Y-shaped poly- propylene mesh (AMS®) - Sacral promontory with 3–4 5 mm tacks (ProTack; Tyco Healthcare, Norwalk, CT, USA) Concomitant surgeries - TVT-O (Gynecare, Ethicon): 14 in LS and 12 in RS (*P* = NS) - Posterior colpoperineorrhaphy: 7 in LS and 4 in RS (*P* = NS]) - Anterior colporrhaphy: 3 in LS and 1 in RS (*P* = NS) - SCH: 36 in LS and 37 in RS (*P* = NS) - BSO: 33 in LS and 37 in RS (*P* = NS) - Transurethral cystoscopy: NS	Primary outcomes: intraoperative bleeding, operative time, and hospitalization Secondary outcomes: surgical complications	40/80 (50.0%)	40/80 (50.0%)	da Vinci Surgical System
12. Mantoo et al. 2013 [37]	France	Prospective databases Single center	118	Pelvic floor disorders	ventral mesh rectopexy: modified D’Hoore rectopexy and levatorpexy - Mesh, trimmed to an L-shaped configuration Concomitant surgeries - TVT: 4 in LS and 3 in RS (*P* = NS)	Function, morbidity and recurrence of symptoms after LS vs RS	74/118 (62.7%)	44/118 (37.3%)	Da Vinci-S surgical system (Intuitive Surgical Inc., Sunnyvale, California, USA)
13. Anger et al. 2014† [38]	USA	Randomized controlled trial Multicenter	78	Symptomatic stage POP II or greater, including significant apical support loss	Sacrocolpopexy two separate pieces of synthetic mesh Concomitant surgeries: - Hysterectomy: 20 in LS and 25 in RS (*P* = 0.252) - Retropubic midurethral sling: 21 in LS and 26 in RS (*P* = 0.488) - Anterior or posterior repair: 4 in LS and 1 in RS (*P* = 0.195)	surgical costs (including costs for robot, initial hospitalization) and re-hospitalization within 6 weeks Secondary outcomes: postoperative pain, POP quantification, symptom severity and quality of life, and adverse events	38/78 (48.7%)	40/78 (51.3%)	NR
14. Joubert et al. 2014 [39]	France	Retrospective Multicenter	56	Obese patients with genital prolapse (BMI > 30 kg/m^2^)	Sacrocolpopexy - Prosthetic macroporous monofilament polypropylene mesh (2 in LS), or a polyester mesh (37 in LS) Mesh location (*P* = < 0.005) - Only anterior: 3 in LS and 0 in RS - Only posterior: 2 in LS and 0 in RS - Both: 34 in LS and 17 in RS Concomitant surgeries - Subtotal Hysterectomy: 13 in LS and 2 in RS (*P* = 0.324) - Midurethral sling: 13 in LS and 11 in RS (*P* = 0.059)	Functional outcomes and complication rates	39/56 (69.6%)	17/56 (30.4%)	three-arm da Vinci® surgical system
15. Nosti et al. 2014 [40]	USA	Retrospective Multicenter	535	NR	Sacrocolpopexy type 1 (monofilament, macroporous) polypropylene - Excluded women underwent hysteropexy and women who had a previous sacrocolpopexy or a vaginal mesh repair	Compare perioperative and postoperative surgical outcomes	273/535 (51.0%)	262/535 (49.0%)	NR
16. Unger et al. 2014 [41]	USA	Retrospective Single center	370	NR	Sacrocolpopexy the mesh including 2 arms of approximately 4 × 15 cm in size Concomitant surgeries - Hysterectomy: 28.7% in LS and 20% in RS (*P* = 0.05) - Rectocele repair: 48.7% in LS and 33.1% in RS (*P* = 0.002)	Compare peri- and postoperative adverse events: LS vs RS whether hysterectomy and rectopexy at the time of sacrocolpopexy were associated with these adverse events	249/370 (67.3%)	121/370 (32.7%)	NR
17. Cucinella et al. 2016 [42]	Italy	Case–control (Retrospective) Multicenter	40	Apical prolapse (Stage III-IV according to POP-Q system)	Sacrocolpopexy non-absorbable polypropylene mesh where the anterior portion of the mesh was shaped to Y	Evaluate the safety, feasibility and non-inferiority of RS compared to LS	20/40 (50.0%)	20/40 (50.0%)	DaVinci Surgical System (Intuitive Surgical Inc., Sunnyvale, California, USA)
18. Kenton et al.2016† [43]	USA	Additional analysis of a randomized comparative effectiveness trial	66	Symptomatic stage ≥ 2 POP	Sacrocolpopexy 2 pieces of ultra-lightweight polypropylene mesh Concomitant surgeries - SCH - posterior colporrhaphy - retropubic midurethral slings	Anatomic, symptom, and QoL in RS vs LS after 1 year	33/66 (50.0%)	33/66 (50.0%)	NR
19. Mueller et al.2016 † [44]	USA	Additional analysis of a randomized comparative effectiveness trial	75	Symptomatic stage ≥ 2 POP	Sacrocolpopexy Concomitant surgeries - Hysterectomy: 63% in LS and 53% in RS (*P* = 0.49) - Retropubic midurethral sling: 65% in LS and 55% in RS (*P* = 0.049) - Anterior or posterior repair: 3% in LS and 11% in RS (*P* = 0.20)	Trocar site appearances 1-year following in RS vs LS after 1 year	36/75 (48.0%)	39/75 (52.0%)	NR
20. Mueller et al.2016 [45]	USA	Retrospective Single center	458	Symptomatic POP	Sacrocolpopexy - Soft polypropylene mesh (Gynemesh; Ethicon, Somerville, NJ; or Restorelle; Coloplast, Minneapolis, MN) - Starting from February 2010, Restorelle (Coloplast) Concomitant surgeries - SCH: 127 in LS and 151 in RS 9 (*P* = 0.011) - Midurethral sling: 122 in LS and 117 in RS (*P* = 0.823) - SO: 96 in LS and 103 in RS (*P* = 0.416)	Report outcomes and complications in RS vs LS	232/458 (50.7%)	226/458 (49.4%)	NR
21. Paek et al.2016 [46]	Korea	Retrospective Multicenter	54	POP with symptomatic stage ≥ 2	Sacrohysteropexy - Self-styled nonabsorbable polypropylene monofilament Gynemesh (Ethicon Endo-surgery, OH, USA) Concomitant surgeries - Hysterectomy: 1 in LS	Compare RS or LS sacrohysteropexy and open sacrohysteropexy in POP	43/54 (79.6%)	11/54 (20.4%)	da Vinci Si Surgical System (Intuitive Surgical, Inc., CA, USA)
22. Pilka et al. 2017 [47]	Czech Republic	Retrospective Single center	64	Symptomatic stage ≥ 2 uterine/vaginal vault prolapse	Sacropexy non-absorbable polypropylene Y mesh Concomitant surgeries - Hysterectomy: 21 in LS and 5 in RS - SO: 23 in LS and 6 in RS - Midurethral sling: 1 in LS - Vaginal repair: 2 in LS	Learning curve experiences and follow-up in RS vs LS	51/64 (79.7%)	13/64 (20.3%)	DaVinci S Surgical System (Intuitive Surgical Inc., Sunnyvale, California, USA)
23. Unger et al. 2017 [48]	USA	Retrospective Single center	398	POP	Sacrocolpopexy 2 Pieces of light-weight polypropylene mesh Concomitant surgeries - Hysterectomy: 28.9% in LS and 19.7% in RS - Anterior repair: 6.6% in LS and 8.5% in RS - Posterior repair: 49.2% in LS and 33.8% in RS - Midurethral sling: 66.4% in LS and 62.0% in RS	Long-Term Effectiveness	256/398 (64.3%)	142/398 (35.7%)	NR
24. Illiano et al. 2019 [49]	Italy	Prospective, noninferiority Randomized Single center	100	POP stage III or IV	Sacrocolpopexy - In cases of previous hysterectomy, 2 rectangular polypropylene meshes - In cases of uterus preservation, the anterior mesh was a Y-shaped	Efficacy in the treatment of high-stage POP in RS vs LS	51/100 (51.0%)	49/100 (49.0%)	DaVinci Xi
25. Thomas et al.2020‡ [50]	USA	Retrospective cohort study with a cross-sectional, prospective follow-up survey Single center	*N* = 526 Survey response rate was 41.7% (final *n* = 246)	POP	Sacrocolpopexy	Long-term POP recurrence	166/246 (67.5%)	80/246 (32.5%)	NR
26. Greene et al. 2021 [51]	USA	Retrospective cross-sectional analysis of National Inpatient Sample (NIS) database Multicenter	17,107	A primary or secondary diagnosis of genital prolapse	Hysterectomy - Included women ≥ 65 years-old - Excluded women with malignancy	Adnexal surgery trends	10,489/17107 (61.3%)	6618/17107 (38.7%)	NR
27. Jaresova et al.2021 [52]	USA	Prospective Multicenter	40	Uterovaginal apical prolapse	Sacrocolpopexy Concomitant surgeries - Hysterectomy (supracervical or total): 13 in LS and 15 in RS - Adnexectomy (unilateral or bilateral): 12 in LS and 15 in RS - Posterior repair (including perineorrhaphy): 6 in LS and 5 in RS - Midurethral sling: 6 in LS and 8 in RS - Transobturator postanal sling: 4 in LS and 0 in RS - Other: 4 in LS and 2 in RS	Primary: compare the maximum angle of Trendelenburg Secondary: total time in Trendelenburg, total time in maximum Trendelenburg, angle of Trendelenburg at apical suspension, and proportion of surgical time in Trendelenburg	20/40 (50.0%)	20/40 (50.0%)	NR
28. Lallemant et al. 2021 [53]	France	Retrospective Multicenter	214	POP-Q stage ≥ 2	Sacrocolpopexy - The anterior and posterior polypropylene meshes Concomitant surgeries - Subtotal hysterectomy: 130 in LS and 33 in RS (*P* = < 0.01) - Total hysterectomy: 0 in LS and 1 in RS (*P* = 1) - SUI surgery: 21 in LS and 5 in RS (*P* = 0.4)	Morbidity and long‐term efficacy in RS vs LS primary outcome: reoperation for a recurrent POP Secondary outcomes: operative time (time for midurethral sling placement not included), intraopera- tive blood loss, postoperative POP recurrence (operated on or not), complications related to the mesh (sacrocolpopexy or midurethral sling), and occurrence of de novo urinary incontinence	160/214 (74.8%)	54/214 (25.2%)	Da-Vinci surgical system (Intuitive Surgical Inc., Sunnyvale, CA, USA)
29. Panico et al.2021 [54]	Italy	Retrospective Multicenter	70	POP-Q stage III/IV for anterior and/or api- cal compartment	Sacrocolpopexy surgeon-tailored polypropylene mesh (Restorelle® XL Coloplast Corp., Minneapolis, MN, USA) Concomitant surgeries - Subtotal hysterectomy + BSO: 40 in LS and 30 in RS	Surgical outcomes in RS vs LS	40/70 (57.1%)	30/70 (42.9%)	Da Vinci Robot Si Surgical System (Intuitive Surgical, Mountain View, CA, USA)
30. Wang et al. 2021 [55]	USA	Cost effectiveness NR	NR	Prolapse and apical prolapse recurrence	Sacrocolpopexy	Cost effectiveness (in US Dollars) over 5 years vs over 10 years in LS vs RS	NR	NR	NR
31. Andiman et al. 2022 [56]	USA	Retrospective National Multicenter	7675	Uterovaginal prolapse	Hysterectomy With Concurrent Sacrocolpopexy Concomitant surgeries - Total hysterectomy: 1,022 (49.2%) in in LS; 1,384 (24.7%) in RS (*P* = NR) - SCH: 1055 (50.8%) in in LS; 4214 (75.3%) in RS (*P* = < 0.001) - Urinary incontinence: 1156 (55.7%) in LS; 2610 (46.6%) in RS (*P* = 0.001) - Other urogynecologic: 1207 (58.1%) in LS; 2088 (37.3%) in RS (*P* = < 0.001) - Salpingo-oophorectomy: 1399 (67.4%) in LS; 3427 (61.2%) in RS (*P* = 0.032) - Other gynecologic: 168 (8.1%) in LS; 252 (4.5%) in RS (*P* = < 0.001) - Other urologic: 104 (5.0%) in LS; 151 (2.7%) in RS (*P* = 0.006) - Gastrointestinal/ colorectal: 97 (4.7%) in LS; 197 (3.5%) in RS (*P* = 0.19) - Lysis of adhesions: 193 (9.3%) in LS; 451 (8.1%) in RS (*P* = 0.28)	Surgical Complications and Hospital Cost (in US Dollars) in LS vs RS	2077/7675 (27.1%)	5598/7675 (72.9%)	NR
32. Clark et al.2022 [57]	USA	Retrospective Single center	142	Prolapse	Sacrocolpopexy lightweight polypropylene mesh Concomitant surgeries - Hysterectomy: 44 (51.2%) in LS; 41 (73.2%) in RS (*P* = < 0.01) • Supracervical: 40 (90.0%) in LS; 33 (80.5%) in RS (*P* = 0.17) - Midurethral sling: 1 (1.2%) in LS; 4 (7.1%) in RS (*P* = 0.06) - Lysis of adhesions: 33 (38.4%) in LS; 14 (25.0%) in RS (*P* = 0.09) - Posterior repair: 9 (10.7%) in LS; 4 (7.14%) in RS (*P* = 0.48) - Anterior repair: 1 (1.2%) in LS; 19 (33.9%) in RS (*P* = 0.64)	Operative time in LS vs RS Secondary: intraoperative complications, mesh complications, anatomic prolapse recurrence, and retreatment	86/142 (60.6%)	56/142 (39.4%)	DaVinci Si or Xi platform (Intuitive Surgical)
33. Özbaşli et al. 2022[58]	Türkiye	Retrospective Single center	68	POP	Sacrocolpopexy EndoFast Reliant™ SCP mesh or the custom-made non-absorbable 5*2-cm polypropylene Y-mesh Concomitant surgeries - Hysterectomy: 35 (67.3%) in LS; 12 (75.0%) in RS (*P* = 759) - Colporrhaphy anterior/posterior: 49 (94.2%) in LS; 16 (100.0%) in RS (*P* = 1.0) - Urinary incontinence surgery: 47 (90.4%) in LS; 16 (100.0%) in RS (*P* = 0.330)	Perioperative outcomes	52/68 (76.5%)	16/68 (23.5%)	da Vinci Xi Surgical System (Intuitive Surgical, Sunnyvale, CA)
34. Texeira et al. 2022[59]	India	Retrospective Single center	20	POP	Sacrocolpopexy Y-shaped pattern mesh	Outcomes and complications	15/20 (75.0%)	5/20 (25.0%)	da Vinci Si
35. Nilsson et al. 2023[60]	USA	Secondary analysis of RCT Multicenter	90	POP	Sacrocolpopexy Concomitant surgeries - Hysterectomy: 44 (67.7%) in LS; 15 (60.0%) in RS (*P* = 0.49) - Midurethral sling: 5 (7.7%) in LS; 1 (4.0%) in RS (*P* = 0.53) - Anterior colporrhaphy: 1 (1.5%) in LS; 0 (0.0%) in RS (*P* = 0.72) - Posterior colporrhaphy: 12 (18.5%) in LS; 0 (0.0%) in RS (*P* = 0.02) - Perineorrhaphy: 10 (15.4%) in LS; 0 (0.0%) in RS (*P* = 0.04) - Levator myorrhaphy: 6 (9.2%) in LS; 0 (0.0%) in RS (*P* = 0.13) - Lysis of adhesions: 13 (20.0%) in LS; 4 (16.0%) in RS (*P* = 0.87) - Mini-lap for uterine removal: 22 (37.3%) in LS; 7 (30.4%) in RS (*P* = 0.56)	Postoperative pain and pain-related outcomes	65/90 (72.2%)	25/90 (27.8%)	NR
36. Shigemi et al. 2023[61]	Japan	Retrospective national database Multicenter	461	POP	Sacrocolpopexy	Describe and compare the postoperative adverse events and re- treatment for recurrence in LS vs RS	409/461 (88.7%)	52/461 (11.3%)	NR

The referred study was stratified by surgical route (laparoscopic or vaginal); however, the authors did not report results in LS vs RS [62]

*BSO* Bilateral salpingo-oophorectomy, *LS* Laparoscopic surgery, *MR* Using magnetic resonance, *NA* Not available, *NR* Not reported, *NS* Not significant for p-value reports, *NS* Not specified, *QoL* Quality of life, *RS* Robot surgery, *SCH* Supra-cervical hysterectomy, *SCP* Sacralcolpopexy, *SO* Salpingo-oophorectomy, *TOT* Transobturator tape, *TVT-O* Inside-out transvaginal obturator suburethral tape, *UK* United Kingdom, *USA* United states of America

^*^In this study, the LS cases and RS cases reported differently in the abstract and main full-text results. In our study, we accepted the main text results

^†^The same trial (Abdominal Colpopexy: Comparison of Endoscopic Surgical Strategies (ACCESS) Trial, a 2-center randomized surgical trial (NCT01124916))

^‡^The authors included the already published data from Unger et al.[41]

In the analysis of study types, the majority were retrospective studies (*n* = 23, 63.9%), followed by randomized controlled trials (RCTs) (*n* = 4, 11.1%). Prospective studies accounted for 8.3% (*n* = 3), while prospective randomized and prospective non-randomized studies each comprised 5.6% (*n* = 2). Additionally, modeling and cost-effectiveness studies each represented 2.8% (*n* = 1). Of the total cases, 50.0% (*n* = 18) were from single-center studies, 38.9% (*n* = 14) were from multicenter studies, and 5.6% (*n* = 2) had no reported center information (NR). Out of the 36 studies, the diagnosis was reported as follows: 5.6% (*n* = 2) did not specify the diagnosis, 2.8% (*n* = 1) reported complex rectocele, 2.8% (*n* = 1) indicated pelvic floor disorders, and 88.9% (*n* = 32) reported pelvic organ prolapse (POP). Sacrocolpopexy was the most commonly performed procedure, accounting for 88.9% of cases (*n* = 32), where rectopexy (*n* = 1, 2.7%), ventral mesh rectopexy (modified D’Hoore rectopexy and levatorpexy) (*n* = 1, 2.7%), sacropexy (*n* = 1, 2.7%), sacrohysteropexy (*n* = 1, 2.7%), and hysterectomy (*n* = 1, 2.7%) were performed. The total number of patients included in the analysis is 29,172. Of these, 15,301 (52.5%) underwent laparoscopy, while 13,871 (47.5%) underwent robot-assisted procedures. The overall laparoscopy-to-robot ratio is approximately 1.10. Notably, in 23 studies, the laparoscopy group had a higher number of patients compared to the robot-assisted group. Out of 25 studies, 75.0% underwent other concomitant surgeries. In contrast, rectopexy, apical sacropexy, sacrohysteropexy, and sacropexy were each performed in 2.9% of cases (*n* = 1 each). Only half of the studies specified the type of robot system utilized, with all reported cases involving the Da Vinci System.

Table 2 presents a comparison of baseline characteristics, intraoperative variables, postoperative variables, assessment of urogynecological conditions, follow-up duration, and complications. Although one study briefly mentioned it, none of the studies fully adhered to the outcomes as recommended by the National Institutes of Health Terminology Workshop for Researchers in Female Pelvic Floor Disorders [14]. Only four (11.1%) studies did not report baseline characteristics of the patients. More than half of the studies, 55.6% (*n* = 20), found no age differences between the groups, while 58.3% (*n* = 21) did not report BMI differences between the laparoscopy and robot-assisted groups. One study (2.8%) reported a conversion from the robot-assisted to the laparoscopic route. Eleven studies (30.5%) indicated conversion to laparotomy, with the majority of these conversions occurring in the laparoscopic group (*n* = 9, 81.8%) compared to the robot-assisted group. The reporting on surgical duration varied across the studies, with different definitions used. Some studies included the entire duration of the surgery, while others excluded the time spent on docking the robot-assisted system. Additionally, some studies reported surgical duration with concomitant surgeries included, while others did not. The Pelvic Organ Prolapse Quantification (POP-Q) scale was used as the assessment method in more than half of the studies. However, 16.7% of the studies did not report the assessment method. The follow-up duration varied widely among the studies, ranging from one week to three years. Additionally, 16.7% of the studies did not report any follow-up information. Six studies (16.7%) reported on mesh erosions, and none of the studies found statistically significant differences between laparoscopy and robot-assisted surgery. These studies included only sacrocolpopexies. However, four of these studies (66.7%) reported a higher incidence of mesh erosions in the robot-assisted group.

**Table 2:** Characteristics of the patients, surgical procedures, outcomes, and complications

Author, year	Comparison of baseline characters*§	Comparison intraoperative variables	Comparison post-operative variables	Assessment	Follow-up	Complications
1. Patel et al. 2009[26]	No differences - Age - BMI - Number of vaginal deliveries - Stage of prolapse - Number of prior prolapse surgery	- EBL: LS < RS - Operative time: LS > RS - Estimated direct OR costs: LS < RS - Estimated direct instrument/ materials costs: LS < RS - Estimated direct anesthesia costs: LS > RS - Estimated direct miscellaneous costs: LS > RS	- LOS: LS > RS - Estimated direct hospital room costs: LS > RS - Estimated total charges: LS < RS - Estimated total costs: LS < RS - Estimated direct costs: LS < RS - Estimated profits: LS < RS	NR	NR	NR
2. White et al. 2009[27]	No differences - Age - BMI	No differences - Operative time - EBL	No differences - Length of stay - Mean VAPS at discharge	- urodynamic studies - POP-Q	3rd and 6th months	⁃ None intraoperative ⁃ None postoperative
3. Judd et al. 2010[28]	NR	- Baseline analysis cost: LS + $1155 = RS - RS disposables cost: $1500-$4500 - LS disposables cost: $500-$3500 - OR time RS: 130–383 min - OR time LS: 97–334 min	LOS: RS = LS (0.5–3.0)	NR	NR	NR
4. Chan et al. 2011[29]	No differences - Age - Number of vaginal deliveries - Number of prior prolapse surgery	- Operating time: RS > LS (*P* = 0.02) - Blood loss: LS > RS (*P* = 0.42)	- LOS: RS > LS (*P* = 0.05) - Hemoglobin drop: RS > LS (*P* = 0.37)	- POP-Q - Urodynamic study (uroflowmetry and dual channel cystometry)	LS (39 ± 17) > RS(16 ± 11)	- Bladdder injury LS > RS (*P* = 0.68) - Ureteric injury: 1 in RS - Post-site hernia: 1 in RS
5. Paraiso et al. 2011[30]	No differences - Age - Parity - BMI - Hormone therapy use Race - Insurance status - Charlson Comorbidity - Index - Current smoker - Prior hysterectomy - Prior pelvic reconstructive surgery	- Adhesiolysis > 45 min: 17 in LS and 14 in RS - Conversion to laparotomy or vaginal approach: 2 in LS and 3 in RS - Sacrocolpopexy time: LS < RS (*P* = < 0.001) - Sacrocolpopexy suturing time: LS < RS (*P* = < 0.001) - Additional procedure time: LS > RS (*P* = 0.14) - Total operating time: LS < RS (*P* = < 0.001) - Anesthesia time: LS < RS (*P* = < 0.001) - Operating room time: LS < RS (*P* = < 0.001) - Surgery cost: RS > LS (*P* = 0.008) - OR cost: RS > LS (*P* = 0.008)	- Hospitalization cost (not significant) - 6th week postoperative case (not significant) - Hospital stay: LS < RS (*P* = 0.17) - VAPS: RS > LS in week 2–3-4–5-6 (*p *values: 0.02, 0.03, 0.02, 0.02, respectively) - NSAID use: RS > LS (*P* = < 0.005) - Narcotic use: RS > LS (*P* = 0.92) - Return to normal activities (not significant)	- Structured urogynecologic history - Activity assessment scale scores (not significant) - POP-Q (not significant) - Quality-of-life (not significant) - Pelvic Floor Distress Inventory-20 - Prolapse Subscale - Colorectal subscale - Urinary subscale - Pelvic floor impact Questionnaire - Prolapse Subscale - Colorectal Subscale - Urinary Subscale - Pelvic Organ Prolapse/Urinary Incontinence Sexual Questionnaire-12 - EQ-5D - EQ-5D Visual Analog Scale - Activity Assessment Scale	- Weekly till 6th week - 6th month - 1st year	- Cystoscopy: 2 in LS and 2 in RS - Enterotomy: 1 in RS - UTI: 3 in LS and 5 in RS - Small bowel obstruction: 2 in RS - Wound infection: 2 in RS - Erosion: 2 in RS - Abdominal wall pain necessitating trigger point injection: 3 in RS - Abscess: 1 in LS and 1 in RS
6. Tan-Kim et al. 2011[31]	No differences - Weight - Race - Prior pelvic surgery Different - Age: LS > RS (*P* = : < 0.01) - Parity: LS > RS (*P* = : < 0.01)	- Operating time: LS < RS (*P* = : < 0.01) - Setup time: LS < RS (*P* = : < 0.01) - Surgery cost:: LS < RS (*P* = < 0.01) - EBL: RS > LS (*P* = 0.85) - Cystotomy: LS > RS - Colpotomy: LS < RS - Suture in bladder with removal: LS > RS - Ileal perforation: LS > RS - Electrolyte imbalance: LS < RS - Blood transfusion: LS < RS - Trocar site cellulitis: LS < RS	- LOS direct costs excluding surgery: LS > RS (*P* = 0.738) - LOS: RS = LS (*P* = 0.48)	POP-Q	LS > RS (*P* = 0.15)	- Recurrent anterior vaginal wall prolapse: RS < LS - Recurrent posterior vaginal wall prolapse: RS < LS - Mesh erosion: RS > LS
7. Wong et al. 2011[32]	No differences - Age - ASA - Prior abdominal surgery Different - BMI: RS > LS (*P* = 0.03)	- Double-mesh implantation RS > LS (*P* = 0.003) - Operating time: RS > LS (*P* = :0.0001) - EBL: LS > RS (*P* = 0.048) - Conversions to laparotomy: LS > RS (*P* = :0.747)	- UTI: LS > RS - Ileus: LS > RS - LOS: LS = RS	- Dynamic defecography or magnetic resonance defecography - Preoperative colonoscopy - Preoperative manometric testing	- 6 weeks - 6 months - Vaginal and anorectal examinations	- No mortalities - No recurrences
8. Antosh et al. 2012[33]	No differences - Age - BMI - Parity - Ethnicity - Menapausal - Previous hysterectomy - Prior pelvic surgery - Prior prolapse surgery - Prior incontinence surgery - Sexually active - POP-Q stage	- Operating room time: LS < RS (*P* = 0.30) - EBL: LS > RS (*P* = 0.003) - Cystotomy: LS < RS (*P* = 1) - Blood transfusion: LS > RS (*P* = 0.17)	- LOS: LS = RS - Objective cure rates: RS < LS (*P* = 0.72) - POP-Q measurement: no stat. significant difference	POP-Q	3 months	- Wound infection/abscess: RS > LS (P = 1) - UTI: RS > LS (*P* = 0.20) - Fever: RS = LS (*P* = 0.46) - Readmission: RS < LS (*P* = 0.26) - Return to operating room: RS > LS (*P* = 1) - Postoperative SUI: RS > LS (*P* = 1) - Mesh erosion: RS > LS (*P* = 1)
9. Pulliam et al. 2012[34]	No differences - Age - BMI - Prior surgeries	- ORT: LS < RS (*P* = 0.5) - Into ORT †: LS < RS (*P* = 0.002) - EBL: LS > RS (*P* = 0.6) - Conversion: LS > RS (*P* = 0.8) - Setup time excluding the concomitant surgeries: LS < RS (*P* = 0.8) - ORT excluding the concomitant surgeries: LS < RS (*P* = 0.9) - EBL excluding the concomitant surgeries: LS > RS (*P* = 0.8)	- LOR: LS = RS (*P* = 0.3) - LOR excluding the concomitant surgeries: LS = RS (*P* = 1) - POP-Q: *P* = NS	⁃ POP-Q	6th weeks	⁃ Complications: intraoperative: LS > RS (*P* = 0.6)
10. Seror et al. 2012[35]	No differences - BMI - Menopausal status - Obstetric history - Clinical complaint at presentation - Urinary incontinence - Previous gynecologic surgery Different - Age: LS > RS (P = 0.05)	- EBL: LS > RS (*P* = 0.03) - Strict operative time‡: LS > RS (*P* = < 0.0001) - Overall operative time: LS > RS (*P* = 0.4) - Conversion: LS > RS (*P* = 0.9)	- Urinary catheter duration: LS > RS (*P* = 0.03) - Class painkiller: LS > RS (*P* = 0.03) - LOR: LS > RS (*P* = 0.5)	- Preoperative PDFI-20 - Follow-up with PDFI-20 - Baden and Walker classification - Bonney maneuver - Distal Marshall test - Pelvic ultrasonography - Urodynamic studies - Urine analysis - Papanicolaou smear	- 1, 3, 6 months and yearly - Median: LS > RS (*P* = 0.05)	- Post-operative complications according to Clavien’s classiWcation: LS > RS (*P* = 0.3) - UTI: LS > RS (*P* = 0.7) - Vaginal erosion: LS > RS (*P* = 0.7) - Dysuria: LS > RS (*P* = 0.3) - Dyspareunia: LS > RS (*P* = 0.7) - Prolapse recurrence: LS < RS (*P* = 0.3) - De novo SUI: LS > RS (*P* = 0.7) - Constipation: LS > RS (*P* = 0.7)
11. Awad et al. 2013[36]	No differences - Age - Parity - BMI - Hypertension - Cardiac disease - Diabetes - Previous hysterectomy - Urodynamic diagnosis of SUI - POP-Q Stage	- EBL: LS > RS (*P* = < 0.001) - Operative time: LS < RS (*P* = 0.34) - Cystotomy: LS > RS - Conversion to laparotomy: LS > RS	LOR: LS > RS (*P* = < 0.02)	- POP-Q - Preoperative multichannel uro- dynamic testing	3rd month	- No recurrent prolapse (POP-Q stage ≥ 2) at 3rd month - Postoperative de novo SIU: LS > RS - Hematoma porst site: RS > LS - Hernia porst site: RS < LS
12. Mantoo et al. 2013[37]	No differences - Age - BMI - Previous abdominal surgery	- Operation time: LS < RS (*P* = 0.002) - EBL: LS > RS (*P* = 0.012) - Single mesh: LS > RS (*P* = NS) - Double mesh: LS + RS (*P* = NS) - Conversation: LS > RS (*P* = NS)	- LOS: LS > RS (*P* = NS) - ODS Score: LS > RS (*P* = 0.004) No differences - Sexually active - Sexual difficulty - Penetration dyspareunia - Intercourse dyspareunia - Vaginal dryness - Wexner scores	- Preoperative physical examination and standard investigations for PFD - ODS score [higher the worse] - Wexner fecal incontinence score - sexual function using a simplified non- validated sexual activity score	16 months	- Early complications: LS > RS (*P* = 0.019) - Recurrence within 6 months: LS = RS - Recurrence at last follow-up: LS > RS (*P* = NS)
13. Anger et al. 2014[38]	No differences - Age - BMI - Parity - Study site - > high school education - Race - Hispanic ethnicity - Household income - Major comorbidities (diabetes, hearth attack, stroke, asthma, emphysema, cancer, stomach ulcer, IBS) - Postmenopausal - Current estrogen therapy (local or systemic) - Previous surgery for UI - Previous surgery for POP - Prior Hysterectomy	- EB: LS > RS (*P* = 0.113) - Total surgery time: LS < RS (*P* = 0.110) - Procedure time: LS < RS (*P* = 0.030) - Cost excluding robots: LS < RS (*P* = 0.160) - Cost including robots: LS < RS (*P* = < 0.001) - left iliac venotomy: LS = RS - cystotomy: LS > RS	- Total 6-week costs excluding robots: LS < RS (*P* = 0.060) - Total 6-week costs including robots: LS < RS (*P* = < 0.001) - At 1st week - ASA: LS < RS (*P* = < 0.05) - SPS scores LS < RS (*P* = < 0.05) No differences - POP-Q - UDI - POPDI - CRADI - UIQ - CRAIQ - POPIQ	- POP-Q - Brinks scale of pelvic muscle strength - SF-36 - EQ-5D - PGI-I - Hunskaar Severity Index - PFDI - PFIQ - PISQ - AAS - CARE - SPS scores - QALYs	12 months	- AE: LS > RS - Small bowel obstruction: LS = RS - Vaginal granulation tissue and suture exposure: LS > RS - Port site herniation: LS > RS - Pulmonary emboli: RS > LS - Atrial fibrillation: LS > RS - Hematemesis: LS > RS
14. Joubert et al. 2014[39]	No differences - BMI - Parity - Pessary use - Pelvic floor rehabilitation - Tobacco use - Previous C-section - Previous hysterectomy - Previous POP surgery - POP stage - Bp (ICS POP-Q) - SUI patent - SUI masked Different - Age LS < RS (*P* = 0.002) - Post-menopausal status: LS > RS (*P* = 0.030)	- Operative time: LS < RS (*P* = 0.253) - Bladder injury: LS = RS - Laparoconversion: LS > RS	LOS: Operative time: LS = RS (P = 0.989)	- Baden and Walker classification - Urine analysis, - A Pap smear - pelvic ultrasonography - Urodynamic exploration	- Postoperative at 6 and 12 - Months, and then every year	- Post-operative de novo functional disorders: LS > RS (*P* = NS) - Constipation: LS > RS - Straining to defecate: LS > RS - Straining to void: LS > RS - Post-operative POP stage (ICS POP-Q): LS = RS (*P* = NS) - Wound infection: LS > RS (*P* = NS) - Douglas pouch haematoma: LS > RS (*P* = NS) - Pelvic abscess: LS > RS (*P* = NS) - Reoperation for immediate complications: LS > RS (*P* = NS) - Reoperation for urinary incontinence: LS > RS (*P* = NS) - Reoperation for mesh exposure: LS > RS (*P* = NS) - Reoperation for recurrent prolapse: LS = RS (*P* = NS) - Global reoperation rate: LS > RS (*P* = NS) - Operative complications using IUGA/ICS classification: LS > RS (*P* = NS)
15. Nosti et al. 2014[40]	No differences - BMI - Ethnicity - CCI - Menopausal Different - Age: LS < RS (*P* = < 0.01) - Smoking status (*P* = 0.01) - Prior abdominal surgery: LS > RS (*P* = < 0.01) - HRT: LS < RS (*P* = < 0.01)	- Length of surgery: LS < RS (*P* = < 0.0001) - EBL: LS = RS (*P* = 0.77) - Cystotomy: LS > RS (*P* = 0.7) - Enterotomy: LS > RS (*P* = 0.54) - Vascular injury: LS < RS (*P* = 1) - Hemorrhage > 500 ml: LS > RS (*P* = 0.21) - Conversion: LS < RS (*P* = 0.01)	- Change in Hb: LS > RS (*P* = 0.004) - LOS: LS = RS (*P* = 0.005)	⁃ POP-Q	8 months	- Anatomic failures: LS < RS (*P* = NS) - DVT/PE: LS > RS (*P* = < 0.01) - Ileus/SBO: LS > RS (*P* = 1) - Wound infection: LS > RS (*P* = 1) - Ventral hernia: LS < RS (*P* = 0.49) - Mesh erosion: LS > RS (*P* = 0.2) - Blood transfusion: LS > RS (*P* = 0.25) - All complications: LS > RS (*P* = < 0.01)
16. Unger et al. 2014[41]	No differences - BMI - Vaginal parity - preoperative prolapse stage - current tobacco use - menopausal - Previous POP surgery Different - Age: LS < RS (*P* = 0.009)	- OR Time: LS > RS (*P* = 0.01) - operative and case times in patients with concomitant hysterectomy: LS < RS (*P* = < 0.001) - EBL ≥ 500 ml: LS < RS (*P* = 0.01) - Conversion to LS: 3 in RS - Conversion: LS > RS (*P* = 0.81) - Bladder injury: LS < RS (*P* = 0.04) - Bowel injury: LS < RS (*P* = 0.36) - Vascular injury: LS = RS (*P* = 0.98)	⁃ LOS: LS > RS (P = 0.71)	NR	195 days (P = NS)	- Wound infection: LS < RS (*P* = 0.50) - Hematoma: LS > RS (*P* = 0.23) - Transfusion: LS > RS (*P* = 0.49) - Pelvic abscess: LS = RS (*P* = 0.98) - DVT/PE: LS > RS (*P* = 0.32) - Ileus: LS > RS (*P* = 0.32) - Bowel obstruction: LS < RS (*P* = 0.21) - Neurologic injury: LS > RS (*P* = 0.88) - Pulmonary: LS < RS (*P* = 0.97) - Cardiac: LS = RS (*P* = 0.98) - Mesh erosion: LS < RS (*P* = 0.62) - Clavien-Dindo Grading System grade 3: LS > RS (*P* = 0.81)
17. Cucinella et al. 2016[42]	- Age: LS > RS - BMI: LS > RS - Previous pregnancy: LS > RS - SUI: LS < RS - Previous hysterectomy • vaginal: LS > RS • laparoscopic: LS < RS	⁃ Operative time: LS < RS (*P* = < 0.05) ⁃ EBL: LS > RS (*P* = < 0.05) ⁃ Conversion: LS > RS	⁃ LOS: LS > RS (*P* = NS)	⁃ POP-Q ⁃ Careful gynecological and urogynecological evaluation with determination of functional symptoms (presence or absence of SUI),	6 months	No recurrence after 6 months No readmissions
18. Kenton et al.2016[43]	No differences - Age - BMI - Race - Previous UI surgery - Previous POP surgery - Previous hysterectomy	NR	NR	- POP-Q - PFDI - PFIQ - PGI-I - UDI - UDI - POPDI - CRADI - UIQ - POPIQ - CRAIQ - PISQ	1 year	No differences - POP-Q - UDI - POPDI - CRADI - UIQ - POPIQ - CRAIQ - PISQ total - Sexually active
19. Mueller et al.2016[44]	No differences - Age - BMI - Race - Previous UI surgery - Previous POP surgery - Previous hysterectomy	NR	- Stony Brook Scar Evaluation Scale¶ at 6th month: LS > RS (*P* = 0.003) - Stony Brook Scar Evaluation Scale¶ at 12th month: LS > RS (*P* = 0.002) - Optimal wound repair at 1 year: LS > RS (*P* = 0.008)	⁃ Stony Brook Scar Evaluation Scale¶	1 year	NR
20. Mueller et al.2016[45]	No differences - Age - White race - BMI - Parity - Prior prolapse procedure - Prior incontinence procedure - Preoperative POP-Q stage 2 - Preoperative POP-Q stage ¾ Different - Prior hysterectomy: LS > RS (*P* = 0.01)	- Conversion: LS > RS (*P* = 0.0457) - EBL: LS > RS (*P* = 0.241) - Time in OR: LS < RS (*P* = 0.000) - Operating time: LS < RS (*P* = 0.000) - Cystotomy: LS > RS (*P* = 0.052) - Bowel injury: LS > RS (*P* = 0.159)	⁃ NR	- POP-Q - UDI-6 - POPDI-8 - CRADI-8 - Total PFDI	Median: 13 weks	- vaginal mesh erosion: RS > LS No differences postoperative - Partial SBO/ileus: LS < RS (*P* = 0.26) - Port site hernia: LS = RS - Reoperation: LS = RS (*P* = 0.39) - POP-Q stage - UDI-6 - POPDI-8 - CRADI-8 - Total PFDI - Partial SBO/ileus - Port site hernia - Reoperation
21. Paek et al.2016[46]	- Age: LS > RS - Parity: LS > RS - BMI: LS < RS - Previous abdominal surgery: LS = RS - Peritoneal adhesion: LS < RS - preoperative POP-Q stage: LS < RS	- operating time: LS < RS - EBL: LS > RS - Intraoperative complication: None	- Hb drop: LS < RS - POD for Foley catheter removal: LS > RS - same day discharge for all	- POP-Q - Urodynamic studies - questionnaire to assess subjective evaluation of related symptoms before and 12 months after surger	30 (range 12–108) months	- Subjective success rates: LS < RS - Objective success rates: LS < RS - Reoperation: LS > RS - postoperative POP-Q stage: LS = RS - Postoperative symptoms: LS > RS - Overactive bladder: LS > RS - Urinary incontinence: LS > RS - Constipation: LS > RS - Dyspareunia: LS > RS - Mesh erosion: none - Voiding dysfunction: none
22. Pilka et al. 2017[47]	- Age: LS < RS - BMI: LS > RS - Previous pregnancy: LS > RS - Previous hysterectomy • Vaginal: LS > RS • Laparoscopic: LS > RS • Abdominal: LS > RS - Previous POP repair: LS > RS - Previous anti- incontinence surgery: LS > RS	- Operation time: LS < RS (*P* = NS) - EBL: LS > RS (*P* = NS) - Conversion: none	- POP-Q examination (*P* = NS)	- POP-Q - Careful gynecological and uro-gynecological evaluation with determination of functional - Symptoms (presence or absence of SUI) - PGI-I - PISQ-IR	LS: 14,1 months RS: 24,2	- PISQ-IR sexually active: LS > RS
23. Unger et al. 2017[48]	- Age: LS < RS - BMI: LS > RS - Vaginal parity: LS = RS - POP stage: LS = RS - Previous POP surgery: LS < RS - Menopause: LS < RS - Tobacco: LS < RS	NR	NR	- POP-Q	LS: 5.8 (1.9–13.5) months RS: 5.8 (2.8–13.8) months	By year 6 - The estimated composite recurrence rates: LS (57%) > RS (49%) - Rates of symptomatic bulge: LS (49%) > RS (44%) - Rates of POP to or beyond the hymen: LS (22%) > RS (11%) - Rates of retreatment for POP: LS (23%) > RS (8%)
24. Illiano et al. 2019[49]	No differences - Age - BMI - Weight of a baby at its birth - Menopause - Previous prolapse or continence surgery - Anterior colporrhaphy - Transobturator sling - Previous hysterectomy (not prolapse-related) - History of recurrent UTIs - Anxiety-depression syndrome - Hypertension - Diabetes - Thyroid disorders - Education level	- Surgical time: RS > LS (*P* = < .001) - EBL: RS < LS (*P* = 0.97)	- LOS: RS < LS (*P* = 0.98)	- POP-Q - Physical urogynecologic examination - A stress test (with and without prolapse reduction) - urodynamic testing - Uroflowmetry - FSFI - Short forms of Urinary Distress Inventory -6 and Inconti- nence Impact Questionnaire-7 - PGI-I - VAPS	- at 1, 3, 6, and 12 months postopera- tively and then annually - Mean: 24.06 (20.8–36.1) months	- Cure rate for apical compartment: RS = LS - Cure rate for apical anterior and posterior: RS > LS (*P* = NS) - Follow-up: RS > LS (*P* = NS) - Total vaginal length: RS = LS (*P* = NS) - Voiding symptoms: RS = LS (*P* = NS) - Storage symptoms: RS > LS (*P* = NS) - Sexually active: RS < LS (*P* = NS) - Sexual dysfunction: RS < LS (*P* = NS) - Anorectal dysfunction symptoms: RS > LS (*P* = NS) - SUI: RS < LS (*P* = NS) - De Novo case: RS = LS (*P* = NS) - Urgency urinary incontinence: RS = LS (*P* = NS) - Qmax (mean ± SD): RS < LS (*P* = NS) - FSFI: RS > LS (P = NS) - IIQ7: RS = LS (*P* = NS) - UDI-6: RS = LS (*P* = NS) - PGI-I Score 1: RS < LS (*P* = NR) - PGI-I Score 2: RS = LS (*P* = NR) - PGI-I Score 3: RS < LS (*P* = NR) - VAPS: RS = LS (*P* = NS) - Mesh erosion: RS < LS (*P* = NS) - Clavien-Dindo postop complications: RS < LS (*P* = NS) - Nausea and vomiting: RS < LS (*P* = NR) - Fever (Grade 1): RS > LS (*P* = NR) - Transfusion: RS < LS (*P* = NR) - Hematoma: RS > LS (*P* = NR) - Mesh erosion: RS < LS (*P* = NR)
25. Thomas et al.2020[50]	NR	NR	NR	- PFDI-20 - PGI-I	0.5 years (2 days to 13.4 years)	- PGI-I - 1: LS > RS - 2: LS > RS - 3: RS > LS - 4: RS > LS - 5: RS > LS - 6: LS > RS - 7: RS > LS - Looking back, would have surgery again: LS > RS - Usually have a bulge or something falling out that you can see or feel in your vaginal area: RS > LS - Usually experience frequent urination: RS > LS - Usually experience urine leakage associated with a feeling of urgency, that is, a strong sensation of needing to go to the bathroom: RS > LS - Usually experience urine leakage related to coughing, sneezing, or laughing: RS > LS - Usually experience pain or discomfort in the lower abdomen or genital region: LS > RS - complications or problems related to surgery: RS > LS - Complications related to recurrent POP: RS > LS - Complications related to mesh exposure: LS > RS
26. Greene et al. 2021[51]	# - Age - Race/ethinicity - Zip code-level household income - Primary payer - Elective admission - Hospital census region - Hospital bed size - Hospital type	- BSO: LS > RS - USO: RS > LS - BS: RS > LS	NR	NR	NR	
27. Jaresova et al.2021[52]	- Age: RS > LS - BMI: RS > LS - Parity:RS = LS - Current or former smoker: RS > LS - Any comorbidity: RS > LS - Hypertension: RS > LS - COPD: RS = LS - Asthma: RS = LS - History of diverticulitis: RS > LS - IBD: RS = LS - Prior hysterectomy: LS > RS > - Prior surgery for prolapse and/or SUI: LS > RS	- EBL: RS > LS - Bowel preparation: RS > LS • Magnesium citrate: RS > LS • Enema: LS > RS • Sodium phosphate solution: LS > RS - Bowel retractor • T’Lift: LS > RS • Stitch: LS > RS • Fan retractor: LS > RS • Laparoscopic grasper: RS > LS • Robot ProGrasp forceps: RS > LS • Deaver:LS > RS • Breisky-Navratil: LS > RS - Maximum angle of Trendelenburg: LS > RS (*P* = 0.02) - Angle of Trendelenburg at apical suspension: LS > RS (*P* = 0.02) - Total time in Trendelenburg: LS > RS (*P* = 0.01) - Total time in maximum Trendelenburg: RS > LS (*P* = 0.56) - Proportion of total surgical time in Trendelenburg (*P* = 0.72) • < 25%: RS > LS • 25% to < 50%: LS = RS • 50% to < 75%: RS > LS • ≥ 75%: LS > RS • Missing: LS > RS - Bladder injury: VS = RS > LS	NR	POP-Q	NR	- No postoperative complications during the immediate postoperative hospital stay,
28. Lallemant et al. 2021[53]	No differences - Age - BMI - Parity - Active smoking - Menopause - Hormonal substitutive treatment - Diabetes mellitus - History of hysterectomy - History of UI surgery - High-grade prolapses (POP-Q ≥ 3) • At least one vaginal compartment • All three vaginal compartments • Preoperative UI	- Operation time: LS < RS (*P* = 0.05) - Anterior and posterior meshes: LS > RS (*P* = 0.1) - EBL ≥ 500 mL: RS > LS (P = 0.06) - Senior surgeon: RS > LS (*P* = < 0.01) - Intraoperative complication • Event ≥ 1: LS > RS (*P* = 1) • Vaginal perforation: LS > RS (*P* = 1) • Bladder perforation: LS > RS (*P* = 1) • Digestive wound: LS > RS (*P* = 1)	NR	- POP-Q - physical examination between 6 and 8 weeks after surgery	33.7 months in LS and 30.5 months in RS (*P* = 0.3)	Immediate postoperative complication • Event ≥ 1: LS > RS (*P* = 1) • Hematoma of the abdominal muscle: LS > RS (*P* = 1) • Acute pyelonephritis: LS > RS (*P* = 1) • Urinary retention: LS > RS (*P* = 1) • Cystitis: LS < RS (*P* = 1) Long-term postoperative complications • Recurrent POP (POP-Q ≥ 2): RS > LS (*P* = 0.0003) • Global reoperation: RS > LS (*P* = 0.02) • Reoperation for POP recurrence: RS > LS (*P* = 0.01) • Mesh-related reoperation vaginal exposure: LS > RS (*P* = 1) • Mesh-related reoperation Infectious spondylodiscitis: LS > RS (*P* = 1) • UI-related reoperation De novo UI surgery: RS > LS (*P* = 0.07) • UI-related reoperation vaginal exposure: RS > LS (*P* = 0.4)
29. Panico et al.2021[54]	No differences - Age - BMI - Menopause - Parity - Previous abdominal surgery - ASA Score ≥ 2 - POP-Q Stage - POP-Q Score	- Operative time: RS > LS (*P* = 0.0001) - EBL: RS > LS (*P* = NS) - NO Intraoperative complications - Postoperative ileus: RS > LS (*P* = NS) - VAS 4 h: RS = LS (*P* = NS) - VAS 12 h: RS = LS (*P* = NS) - VAS 24 h: RS = LS (*P* = 0.0001) - Cosmetic outcome, patient ##: RS < LS (*P* = 0.0001) - Cosmetic outcome, surgeon ##: RS < LS (*P* = 0.0001)	- LOR (*P* = NS)	- POP-Q - Detailed history - Physical examination - Urodynamic evaluation	1 month	NR
30. Wang et al. 2021 [55]	NR	In 5-year time horizon - Cost: LS/LS for recurrence ($20,037) < RS/RS for recurrence ($23,726) - QALYs: LS/LS for recurrence = RS/RS for recurrence - ICER in reference to expectant management: LS/LS for recurrence (17,795) < RS/RS for recurrence (21,071) - ICER in reference to lowest cost surgical option: both dominated In 10-year time horizon - Cost: LS/LS for recurrence ($20,214) < RS/RS for recurrence ($23,930) - QALYs: LS/LS for recurrence = RS/RS for recurrence - ICER in reference to expectant management: LS/LS for recurrence (9,317) < RS/RS for recurrence (11,030) - ICER in reference to lowest cost surgical option: both dominated	NR	- QALYs - ICER	NR	NR
31. Andiman et al. 2022[56]	No differences - Age - Hypertension - Hypothyroidism - Diabetes - COPD - Depression - Obesity - Income quartile - Insurance payer - Hospital teaching status - Hospital control No differences - Year of surgery (*P* = 0.004) - Hospital bed size (*P* = < 0.001)	NR	NR	NR	NR	- Cost of the index admission: LS < RS (*P* = < 0.001) - 30 day readmission rate: LS > RS (*P* = 0.07) - Any complication: LS > RS (*P* = < 0.001) - Any major complication: LS > RS (*P* = < 0.001) • Cardiovascular event: LS > RS (*P* = 0.84) • Respiratory failure: LS > RS (*P* = 0.25) • Renal failure: LS > RS (*P* = 0.13) • UTI: LS > RS (*P* = < 0.001) • Surgical bleeding/vessel injury: LS > RS (*P* = 0.001) • Accidental laceration or retained foreign body: LS > RS (*P* = < 0.01) • Transfusion: LS > RS (*P* = 0.49) • Ileus/obstruction: LS > RS (*P* = 0.37) • Other: LS > RS (*P* = 0.015) - Any minor complication: LS > RS (*P* = 0.27) • Nausea/vomiting: LS > RS (*P* = 0.27) • Pneumonia: LS > RS (*P* = 0.42) • Other respiratory complication: LS > RS (*P* = 0.17) • Incisional complication: LS = RS (*P* = 0.99) • UTI: LS < RS (*P* = 0.80)
32. Clark et al.2022[57]	No differences - Age - BMI - Race - Parity - Diabetes - Smoking/tobacco use - Preoperative prolapse stage - Surgeons with < 10 yrs experience Difference - Resident involvement: LS > RS (*P* = 0.01)	- Operative Time: RS < LS (*P* = 02) - Operative Time with concomitant hysterectomy: RS < LS (*P* = 02) - RS Operative Time: with dedicated surgery team < without dedicated surgery team (*P* = < 0.01)	NR	NR	Mean: 127: LS < RS (*P* = 0.16)	- intraoperative bladder or bowel injury: LS > RS (*P* = 0.15) - anatomic recurrence beyond the hymen: LS < RS (*P* = 0.09) - retreatment with pessary or reoperation: LS < RS (*P* = 0.18) - postoperative mesh complications: LS < RS (*P* = 0.39)
33. Özbaşli et al. 2022[58]	No differences - Age - BMI - Parity - Menopause - Menopause duration (years) - HRT - Concomitant disease - Number of vaginal births - Previous hysterectomy - Previous surgery for urinary incontinence - Previous surgery for POP	- Drain: LS > RS (*P* = 1.0) - EndoFast Reliant™: RS > LS (*P* = 0.542) - Number of sutures used to suture the mest to the vagina: *P* = 964 - Operative time: RS > LS (*P* = < 0.001)	- LOS: RS > LS (*P* = 0.565) - Pre-/postoperative hemoglobin difference: LS > RS (*P* = 0.949) - Duration of urinary catheter: RS > LS (*P* = 0.371) - Pain score: LS > RS (*P* = 0.256)	NR	3 years	- Intraoperative complications: none - Early postoperative complications: RS > LS (*P* = 0.015) - Readmission: RS > LS (*P* = 0.712) - Late postoperative complications: RS > LS (*P* = 0.620) - Secondary surgery for complications: LS > RS (*P *= 1.0) - Timing of postoperative complications: *P* = 0.120 - Ileus and re-operation: LS > RS
34. Texeira et al.2022[59]	NR	- EBL: LS > RS - Operative time: RS > LS - Bladder injury: LS > RS - Urethra injury: LS > RS	- Transfusion:: LS > RS - LOS: RS < LS	NR	1 year	- Clavien-Dindo grade I: RS > LS - Clavien-Dindo grade II: RS > LS - Clavien-Dindo grade IIIa: RS < LS - Recurrence: LS > RS
35. Nilsson et al. 2023[60]	No differences - Age - BMI - Race - Postmenopausal - Current smoker - Parity - Baseline POP-Q Stage - Baseline VAPS - Preoperative IV acetaminophen	- Amount of local anesthesia - EBL - IV fluids - Operating room time	- LOS: LS > RS (*P* = 0.02) - Change from baseline in VAS pain score at 24 h: LS < RS (*P* = 0.71) - Univariate analysis VAPS: no difference (*P* = 0.71) - After adjusting for confounders: no difference (*P* = 0.79) - MME: LS > RS (*P* = 0.12) - 24 h VAPS without concomitant surgeries: LS > RS (*P* = 0.82) - MME univariable analysis: LS > RS (*P* = 0.02) - MME univariable analysis after accounted for length of surgery: no difference (*P* = 0.61) - Opioid requirements: • First 24 h (including intraoperative): LS > RS (*P* = < 0.01) • First 24 h (excluding intraoperative): LS > RS (*P* = 0.12) • 7-d diary opioid use after discharge: LS > RS (*P* = 0.19)	- POP-Q - MME - VAPS - PROMIS PI-SF-8a - One-week pain diaries	1 week	- Intraoperative complications - PROMIS PI-SF-8a: RS > LS (*P* = 0.94)
36. Shigemi et al. 2023[61]	No differences - Age - Asthma - Hypertension - Diabetes mellitus - Dyslipidemia - Depression and anxiety-related diseases - Delirium - Cystocele - Uterine prolapse - Rectocele - POP with unknown details - Urinary incontinence - Dysuria	NR	NR	NR	Total observation: LS > RS (*P* = 0.241) Postoperative follow-up: LS > RS (*P* = < 0.001)	- Postoperative adverse events • Composite outcome: LS > RS (*P* = 0.270) • Vaginal, wh: LS < RS • Postoperative urinary incontinence: LS > RS • Postoperative dysuria: LS > RS • Abdominal incisional hernia: LS > RS - Re-treatment for recurrence • Composite outcome: LS < RS (*P* = 0.113) • Surgery: LS < RS

*AAS* Activities assessment scale, *AE* Adverse effect, *ASA* American society of anesthesiologists classification, *BMI* Body mass index, *BS* Bilateral salpingectomy, *BSO* Bilateral salpingo-oophorectomy, *CARE* Convalescence and recovery evaluation, *CCI* Charleston co-morbidity Index, *CRADI* Colon rectal anal distress inventory, *CRAIG* Colon rectal anal impact questionnaire, *DVT* Deep vein thrombosis, *EBL* Estimated blood loss, *EQ-5D* EuroQol-5D, *FISI* Fecal incontinence severity index, *FSFI* Female sexual function index, *Hb* Hemoglobin, *HRT* Hormone replacement therapy, *IBS* Irritable bowel syndrome, *ICER* Incremental cost-effectiveness ratio, *ICS* International continence society, *LOS* Length of hospital stay in days, *LS* Laparoscopic surgery, *MMEs* Morphine milligram equivalents, *NA* Not available, *NR* Not reported, *NS* Not specified, *ODS* Obstructed defaecation syndrome, *OR* Operating room, *ORT* Operating room time, *P* p-value, *PDFI* Pelvic floor distress inventory, *PFD* Pelvic floor disorders, *PFDI* Pelvic floor distress inventory, *PFIQ* Pelvic floor impact questionnaire, *PISQ* Pelvic organ prolapse/urinary incontinence sexual questionnaire, *PISQ-IR* Pelvic organ prolapse/urinary incontinence sexual questionnaire, *PGI-I* Patient global impression of improvement, *POP* pelvic organ prolapse, *POPDI* Pelvic organ prolapse distress inventory, *POPIQ* Pelvic organ prolapse impact questionnaire, *POP-Q* Pelvic organ prolapse quantification scale, *PROMIS PI-SF-8a* Patient-reported outcomes measurement information system − pain interference short form, *POD* Postoperative day, *QALYs* Quality-adjusted life years, *RS* Robot surgery, *SBO* Small bowel obstruction, *SF-36* Short form health survey, *SPS* surgical pain scales, *SUI* Stress urinary incontinence, *UDI* Urinary distress inventory, *UI* Urinary incontinence, *UIQ* Urinary impact questionnaire, *USO* Unilateral salpingo-oophorectomy, *UTI* Urinary tract infection, *VAPS* Visual analog pain scale

^†^Operating room (OR) time, defined as the interval from when the patient enters the operating room to the beginning of the surgical procedure

^‡^Strict operative time: defined as excluding time for preparation and docking of robot and including port placement

^§^If not specified, conversion means to laparotomy

^¶^The higher is the better

^#^The results were adjusted and included missing data

^##^The larger is the better

**Fig. 4:**
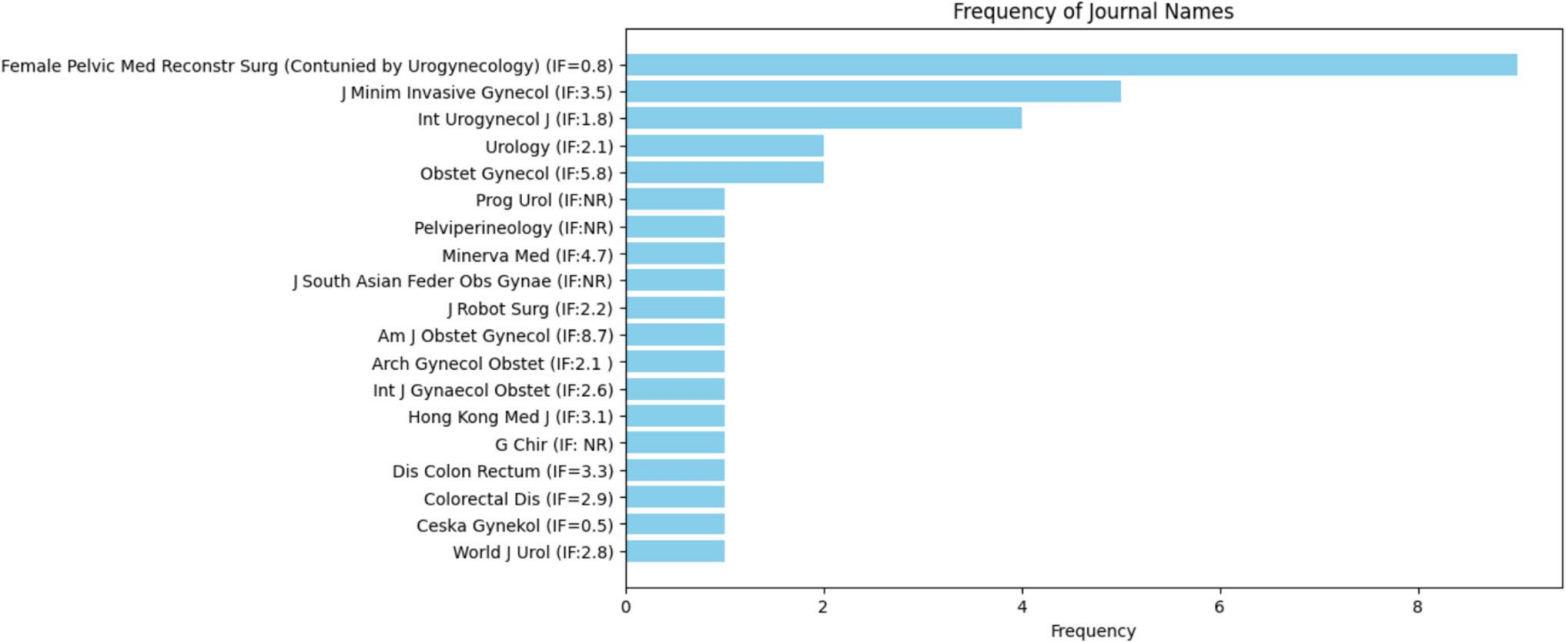
Journal names and their impact factors: a bar chart displaying the journals that published the included studies along with their respective impact factors

**Fig. 5:**
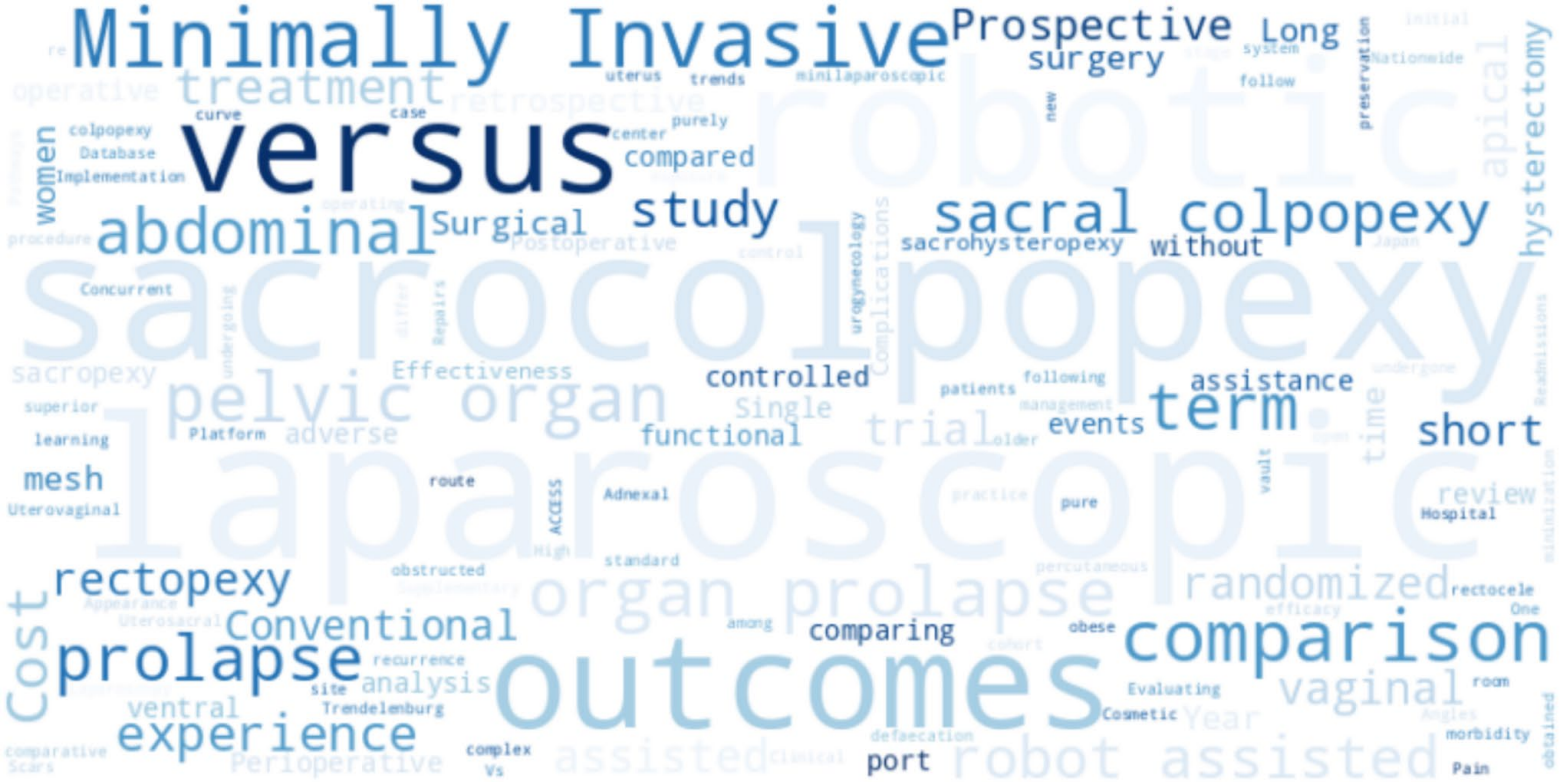
Word cloud of study titles: a visual representation of the frequency of the words in the titles of the included studies, with font sizes reflecting their frequency

The frequencies in the journal were represented in Fig. 4, where Female Pelvic Medicine & Reconstructive Surgery (continued by Urogynecology) was the most common journal, accounting for 25.0% (*n* = 9) followed by J Minim Invasive Gynecol (*n* = 5, 13.9%), Int Urogynecol J (*n* = 4, 11.1%). The impact factors of the journals ranged from a minimum of 0.5 to a maximum of 8.7. The word cloud analysis, shown in Fig. 5, highlights sacrocolpopexy, laparoscopy, and robots as the most frequent terms.

A moderate positive correlation (*r* = 0.498) was observed between journal impact factor and total citations, indicating that journals with higher IF values generally tend to accumulate more citations, although the relationship is not strong and suggests that additional factors also influence citation counts.

**Fig. 6:**
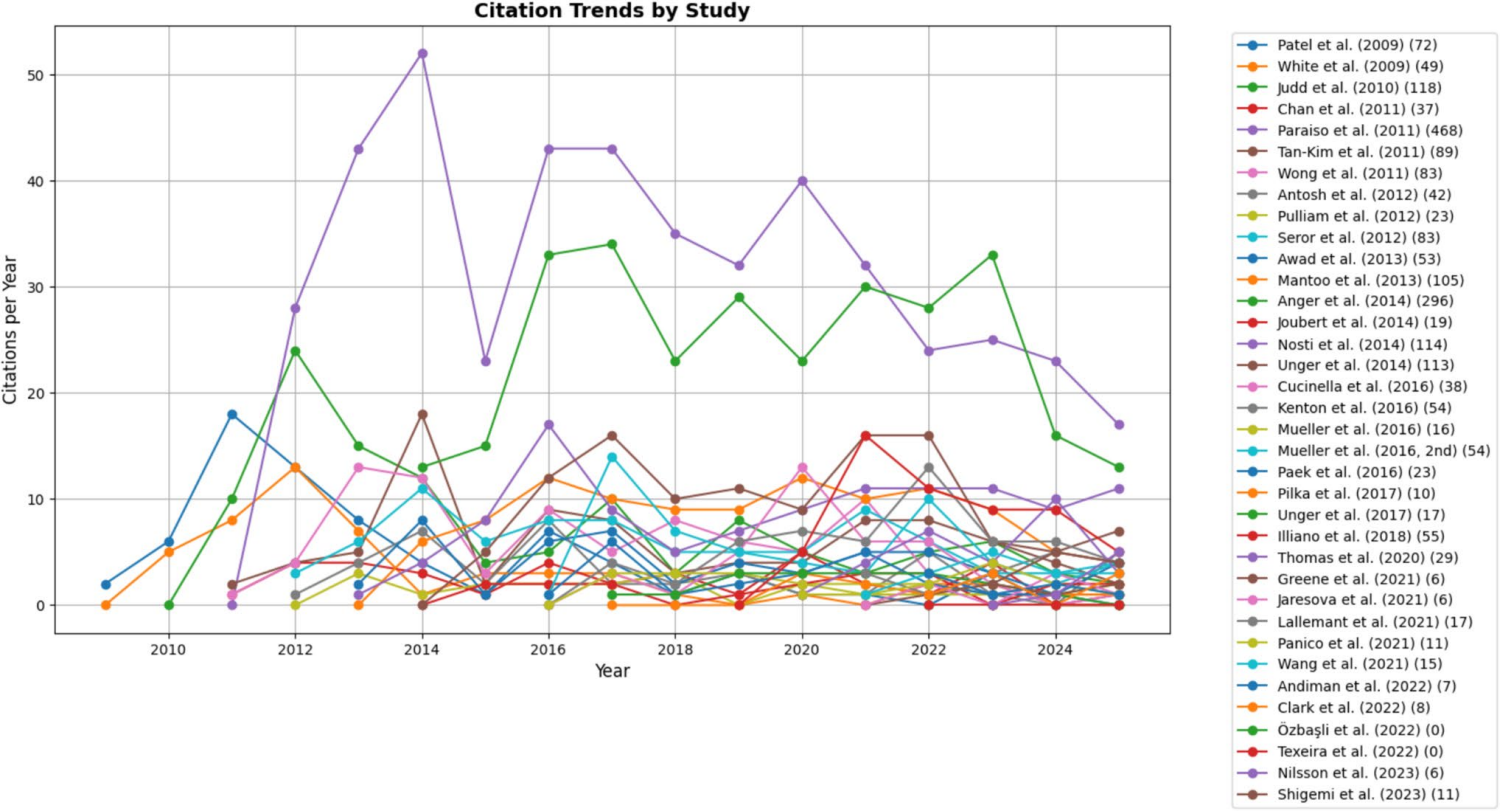
Longitudinal citation dynamics of included studies. Yearly citation counts are plotted for each study, with the total cumulative citations shown in parentheses next to the study label (Author et al. (year) (total citations)).

Furthermore, we illustrated the citation trends of each study over time in Fig. 6. The total number of citations ranged from 0 to 468. Citation trajectories generally followed a typical pattern, with most studies reaching a peak within 2–5 years of publication before showing a gradual decline. A subset of studies maintained higher citation rates over time, reflecting their broader influence in the field. In contrast, more recent studies had relatively low citation counts, likely due to limited time for dissemination rather than lack of impact. Notably, the highest citation counts were observed for randomized controlled trials.

## Discussion

Our final analysis included 36 studies published between 2009 and 2023, primarily from high-income countries. Most studies were retrospective (63.9%), followed by randomized controlled trials (11.1%). POP was the most common diagnosis (88.9%), and sacrocolpopexy was the most performed procedure (88.9%). Of 29,452 patients, 15,301 underwent laparoscopy and 13,871 robot-assisted surgery, with a laparoscopy-to-robot-assisted ratio of 1.10. Most studies (75%) included concomitant surgeries, with 16.7% reporting mesh erosions, predominantly in the robot-assisted group. However, mesh erosions cannot be directly attributed to robot-assisted surgery, as there are numerous confounding variables across the studies. Therefore, the data cannot be pooled to conclude that laparoscopy is safer than robot-assisted surgery in terms of mesh erosion risk. Nevertheless, this remains an observational finding from our analysis and should be validated through prospective randomized studies. Variations in reporting and follow-up durations were observed. The journal Female Pelvic Medicine and Reconstructive Surgery was the most common publication source. A moderate correlation was observed between journal impact factor and total citations, with citation trajectories typically peaking within 2–5 years and randomized controlled trials showing the highest sustained impact.

A decade of data highlights the efficacy and safety of robot-assisted sacrocolpopexy, showcasing its benefits over abdominal sacrocolpopexy [17]. Robot-assisted sacrocolpopexy is associated with reduced blood loss, less postoperative pain, and a faster recovery time [17]. It also offers a shorter learning curve when compared to laparoscopic sacrocolpopexy [17]. A meta-analysis encompassing 13 studies with a total of 2,115 participants demonstrated comparable efficacy between robot-assisted and laparoscopic sacrocolpopexy [18]. Although robot-assisted sacrocolpopexy was associated with reduced blood loss and a lower conversion rate, these differences were not regarded as clinically significant [18]. Although our study showed a higher conversion rate in the robot-assisted group, this difference was not statistically significant. A systematic review and meta-analysis comparing robot-assisted and laparoscopic ventral mesh rectopexy found that, contrary to previous reports, the robot-assisted surgery did not result in longer operating times [19]. Additionally, this study observed a shorter hospital stay for patients undergoing robot-assisted surgery, with no significant differences in other postoperative outcomes [19]. However, further data, including cost-effectiveness analyses, are needed to determine whether the use of the robot-assisted platform is justifiable [19]. A systematic review including 35 studies on best practices for the perioperative management of abdominal sacrocolpopexy found that laparoscopic sacrocolpopexy was linked to a shorter postoperative hospital stay with no significant difference [20].

A video article published in 2015 critically analyzes the use of robot-assisted surgery in a 26 min laparoscopic sacral colpopexy procedure [21]. In this case, it is important to consider the importance of considering learning curves associated with robot-assisted versus laparoscopic surgeries, as well as the impact of docking time in robot-assisted surgery. Additionally, patient selection is highlighted as a crucial factor in determining the success and efficiency of these procedures. A retrospective analysis utilizing the American College of Surgeons National Surgical Quality Improvement Program (ACS-NSQIP) database raises questions about the role of laparoscopy in the age of robot-assisted surgery [22]. The analysis suggests that, by 2025, robot-assisted surgery may surpass both laparoscopy and open surgery in performing colectomies, proctectomies, pancreatectomies, and esophagectomies [22]. In our study, we also observed that robot-assisted surgeries were more frequent than laparoscopic procedures.

Our study has several strengths, including a comprehensive search strategy across multiple databases in adherence to PRISMA guidelines. The clear and focused research question on urogynecologic surgeries using robot-assisted surgery versus laparoscopy, combined with specific inclusion criteria, minimized bias and ensures data relevance. On the other hand, one notable limitation of our study is the inclusion of multiple types of urogynecologic surgeries rather than focusing on a single standardized procedure. This heterogeneity may have contributed to the variability in outcomes and complicates direct comparisons, potentially limiting the generalizability and clarity of our results. The use of Python for statistical analysis with a clear significance threshold adds to the robustness of the study. However, there are several limitations. The exclusion of certain surgical techniques, such as vaginal natural orifice transluminal endoscopic surgery, mini-laparoscopy, and single-port surgeries, may limit the study’s scope. The restriction to English-language full-text articles could introduce language bias. As none of the included studies adhered to the standardized outcome definitions proposed by the National Institutes of Health Terminology Workshop for Researchers in Female Pelvic Floor Disorders, our analysis was limited by the substantial variability in how outcome variables were reported. [14] This highlights the importance of adhering to standardized outcome reporting, which is essential for improving the quality of research in urogynecology. Such adherence would enable more reliable meta-analyses and contribute to higher-quality systematic reviews. The heterogeneity of the selected studies regarding reported intraoperative and postoperative variables, patient characteristics, follow-up strategies, outcomes, and complications represented a major limitation, restricting more complex comparisons between the laparoscopy and robot surgery groups.

Further investigation into the cost-effectiveness, patient selection criteria, and long-term outcomes is crucial to defining the role of robot-assisted surgery in urogynecology. Future research should focus on comparing robot-assisted surgery with traditional laparoscopic methods, especially in procedures like sacrocolpopexy. Long-term outcomes, cost–benefit analyses, and the impact of learning curves and patient selection on surgical success must be prioritized. Policymakers should also explore strategies for integrating robot platforms efficiently and equitably into healthcare systems, particularly in LMICs, where access to such technologies is limited. Furthermore, all the procedures reported in our study were performed using the Da Vinci System. Future research should compare various robot systems, including the Dexter Robot System™ (Distalmotion, Switzerland), which enhances direct interaction with the surgical team, and the Versius® surgical robot system, which offers advantages in haptic feedback and cost reduction through standard reusable instruments [23, 24]. This preliminary report of 60 robot-assisted sacrocolpopexy cases using the new HUGO RAS system demonstrated its feasibility, safety, and efficacy, with high anatomic (96.7%) and subjective (98.3%) success rates and minimal complications, supporting its potential for broader clinical adoption [25].

Future research should not only compare robot-assisted surgery with traditional laparoscopic methods in terms of long-term outcomes, cost-effectiveness, and patient selection but also address the barriers to conducting and implementing such research in LMICs, where limited infrastructure, high acquisition and maintenance costs, and lack of specialized training restrict access. Strategies, such as international collaborations, structured training exchanges, and cost-reduction initiatives, are needed to enhance global representation and equity. Moreover, as all procedures in our study were performed with the Da Vinci System, future investigations should also include comparative analyses of emerging robot platforms, such as the Dexter Robot System™, the Versius® system, and the HUGO™ RAS system, each offering unique advantages in ergonomics, usability, cost reduction, and clinical outcomes, which may significantly influence the future adoption of robot surgery worldwide.

Our study highlights the minor research from LMICs, emphasizing the need for further investigation into minimally invasive approaches in diverse healthcare settings.

## Conclusion

Our study, which includes data from 36 studies encompassing 29,452 patients, underscores the growing prevalence of robot-assisted surgery, especially for sacrocolpopexy. However, variability in reporting and follow-up durations highlight the need for more standardized protocols to overcome the limitations due to high heterogeneity of the studies.

## Supplementary Information

Below is the link to the electronic supplementary material.

Supplementary file1 (DOCX 21 KB)

Supplementary file2 (IPYNB 1329 KB)

## Data Availability

No datasets were generated or analyzed during the current study.
