# Supplementary material for: A systematic review and bibliometric analysis of robot vs. laparoscopic surgery in urogynecology: current trends and future directions
Source: J Robot Surg. 2025 Nov 3;19(1):748. doi: 10.1007/s11701-025-02885-2 (PMC12583385; doi:10.1007/s11701-025-02885-2)
Supplement: Supplementary file 1 — Supplementary file1 (DOCX 21 KB) [file 11701_2025_2885_MOESM1_ESM.docx]

**Pubmed**

4 (("Robot-Assisted Surgery" OR Robot OR "Robotic Surgery" OR "Da Vinci" OR Aesop OR Zeus OR "Robotic Surgical Procedures"[Mesh] OR "Robot-Assisted Surgeries" OR "Robot-Assisted Surgery" OR "Robotic-Assisted Surgery" OR "Robot-Enhanced Surgery" OR "Surgery, Computer-Assisted"[Mesh] OR "Computer-Assisted Surgeries" OR "Computer-Assisted Surgery" OR "Computer Assisted Surgery") AND (laparoscopic OR "laparoscopic techniques" OR laparoscopy OR "laparoscopic procedure" OR "laparoscopic surgical treatment" OR "Laparoscopy"[Mesh] OR Laparoscopies OR "Laparoscopic Surgical Procedure" OR "Laparoscopic Surgery")) AND (Burch OR Colposuspension OR Fistula OR Urethropexy OR "Pelvic organ prolapse" OR Prolapse OR Sacrohysteropexy OR Sacrocolpopexy OR Cervicosacropexy OR CSP OR "Sacral Colpopexy" OR Enterocele OR Cystocele OR Incontinence OR Incontinent OR Band OR Type 19- Repair OR Pectopexy OR Cervicopectopexy OR Colpopectopexy OR "Native tissue repair" OR "Anterior colporrhaphy" OR "Anterior vaginal wall repair" OR "Posterior colporrhaphy" OR "Posterior vaginal wall repair") Most Recent ("Robot-Assisted Surgery"[All Fields] OR ("robot"[All Fields] OR "robot s"[All Fields] OR "robotically"[All Fields] OR "robotics"[MeSH Terms] OR "robotics"[All Fields] OR "robotic"[All Fields] OR "robotization"[All Fields] OR "robotized"[All Fields] OR "robots"[All Fields]) OR "Robotic Surgery"[All Fields] OR "Da Vinci"[All Fields] OR "Aesop"[All Fields] OR "Zeus"[All Fields] OR "Robotic Surgical Procedures"[MeSH Terms] OR "Robot-Assisted Surgeries"[All Fields] OR "Robot-Assisted Surgery"[All Fields] OR "Robotic-Assisted Surgery"[All Fields] OR "Robot-Enhanced Surgery"[All Fields] OR "surgery, computer assisted"[MeSH Terms] OR "Computer-Assisted Surgeries"[All Fields] OR "computer-assisted surgery"[All Fields] OR "computer assisted surgery"[All Fields]) AND ("laparoscopes"[MeSH Terms] OR "laparoscopes"[All Fields] OR "laparoscope"[All Fields] OR "laparoscopical"[All Fields] OR "laparoscopically"[All Fields] OR "laparoscopics"[All Fields] OR "Laparoscopy"[MeSH Terms] OR "Laparoscopy"[All Fields] OR "laparoscopic"[All Fields] OR "laparoscopic techniques"[All Fields] OR ("laparoscopie"[All Fields] OR "Laparoscopy"[MeSH Terms] OR "Laparoscopy"[All Fields] OR "laparoscopies"[All Fields]) OR "laparoscopic procedure"[All Fields] OR "laparoscopic surgical treatment"[All Fields] OR "Laparoscopy"[MeSH Terms] OR ("laparoscopie"[All Fields] OR "Laparoscopy"[MeSH Terms] OR "Laparoscopy"[All Fields] OR "laparoscopies"[All Fields]) OR "Laparoscopic Surgical Procedure"[All Fields] OR "Laparoscopic Surgery"[All Fields]) AND ("burch"[All Fields] OR "burch s"[All Fields] OR ("colposuspension"[All Fields] OR "colposuspensions"[All Fields]) OR ("fistula"[MeSH Terms] OR "fistula"[All Fields] OR "fistulas"[All Fields] OR "fistula s"[All Fields] OR "fistulae"[All Fields] OR "fistulaes"[All Fields]) OR ("urethropexies"[All Fields] OR "urethropexy"[All Fields]) OR "Pelvic organ prolapse"[All Fields] OR ("prolapse"[MeSH Terms] OR "prolapse"[All Fields] OR "prolapses"[All Fields] OR "prolapsed"[All Fields] OR "prolapsing"[All Fields]) OR "Sacrohysteropexy"[All Fields] OR ("sacrocolpopexies"[All Fields] OR "sacrocolpopexy"[All Fields]) OR "Cervicosacropexy"[All Fields] OR ("crit soc policy"[Journal] OR "contemp secur policy"[Journal] OR "contemp sch psychol"[Journal] OR "conserv sci pract"[Journal] OR "cad saude colet"[Journal] OR "csp"[All Fields]) OR "Sacral Colpopexy"[All Fields] OR ("hernia"[MeSH Terms] OR "hernia"[All Fields] OR "enterocele"[All Fields] OR "enteroceles"[All Fields]) OR ("cystocele"[MeSH Terms] OR "cystocele"[All Fields] OR "cystoceles"[All Fields] OR "cystocoele"[All Fields] OR "cystocoeles"[All Fields]) OR ("incontinance"[All Fields] OR "incontinence"[All Fields] OR "incontinences"[All Fields] OR "incontinency"[All Fields] OR "incontinent"[All Fields] OR "incontinents"[All Fields]) OR ("incontinance"[All Fields] OR "incontinence"[All Fields] OR "incontinences"[All Fields] OR "incontinency"[All Fields] OR "incontinent"[All Fields] OR "incontinents"[All Fields]) OR ("band"[Journal] OR "band"[All Fields]) OR ("Type"[All Fields] AND "19 lond"[Journal] AND ("repairability"[All Fields] OR "repairable"[All Fields] OR "repaire"[All Fields] OR "repaired"[All Fields] OR "repairment"[All Fields] OR "wound healing"[MeSH Terms] OR ("wound"[All Fields] AND "healing"[All Fields]) OR "wound healing"[All Fields] OR "repair"[All Fields] OR "repairing"[All Fields] OR "repairs"[All Fields])) OR "Pectopexy"[All Fields] OR "Cervicopectopexy"[All Fields] OR "Colpopectopexy"[All Fields] OR "Native tissue repair"[All Fields] OR "Anterior colporrhaphy"[All Fields] OR "Anterior vaginal wall repair"[All Fields] OR "Posterior colporrhaphy"[All Fields] OR "Posterior vaginal wall repair"[All Fields]) 2,568

3 Burch OR Colposuspension OR Fistula OR Urethropexy OR "Pelvic organ prolapse" OR Prolapse OR Sacrohysteropexy OR Sacrocolpopexy OR Cervicosacropexy OR CSP OR "Sacral Colpopexy" OR Enterocele OR Cystocele OR Incontinence OR Incontinent OR Band OR Type 19- Repair OR Pectopexy OR Cervicopectopexy OR Colpopectopexy OR "Native tissue repair" OR "Anterior colporrhaphy" OR "Anterior vaginal wall repair" OR "Posterior colporrhaphy" OR "Posterior vaginal wall repair" Most Recent "burch"[All Fields] OR "burch s"[All Fields] OR ("colposuspension"[All Fields] OR "colposuspensions"[All Fields]) OR ("fistula"[MeSH Terms] OR "fistula"[All Fields] OR "fistulas"[All Fields] OR "fistula s"[All Fields] OR "fistulae"[All Fields] OR "fistulaes"[All Fields]) OR ("urethropexies"[All Fields] OR "urethropexy"[All Fields]) OR "Pelvic organ prolapse"[All Fields] OR ("prolapse"[MeSH Terms] OR "prolapse"[All Fields] OR "prolapses"[All Fields] OR "prolapsed"[All Fields] OR "prolapsing"[All Fields]) OR "Sacrohysteropexy"[All Fields] OR ("sacrocolpopexies"[All Fields] OR "sacrocolpopexy"[All Fields]) OR "Cervicosacropexy"[All Fields] OR ("crit soc policy"[Journal] OR "contemp secur policy"[Journal] OR "contemp sch psychol"[Journal] OR "conserv sci pract"[Journal] OR "cad saude colet"[Journal] OR "csp"[All Fields]) OR "Sacral Colpopexy"[All Fields] OR ("hernia"[MeSH Terms] OR "hernia"[All Fields] OR "enterocele"[All Fields] OR "enteroceles"[All Fields]) OR ("cystocele"[MeSH Terms] OR "cystocele"[All Fields] OR "cystoceles"[All Fields] OR "cystocoele"[All Fields] OR "cystocoeles"[All Fields]) OR ("incontinance"[All Fields] OR "incontinence"[All Fields] OR "incontinences"[All Fields] OR "incontinency"[All Fields] OR "incontinent"[All Fields] OR "incontinents"[All Fields]) OR ("incontinance"[All Fields] OR "incontinence"[All Fields] OR "incontinences"[All Fields] OR "incontinency"[All Fields] OR "incontinent"[All Fields] OR "incontinents"[All Fields]) OR ("band"[Journal] OR "band"[All Fields]) OR ("Type"[All Fields] AND "19 lond"[Journal] AND ("repairability"[All Fields] OR "repairable"[All Fields] OR "repaire"[All Fields] OR "repaired"[All Fields] OR "repairment"[All Fields] OR "wound healing"[MeSH Terms] OR ("wound"[All Fields] AND "healing"[All Fields]) OR "wound healing"[All Fields] OR "repair"[All Fields] OR "repairing"[All Fields] OR "repairs"[All Fields])) OR "Pectopexy"[All Fields] OR "Cervicopectopexy"[All Fields] OR "Colpopectopexy"[All Fields] OR "Native tissue repair"[All Fields] OR "Anterior colporrhaphy"[All Fields] OR "Anterior vaginal wall repair"[All Fields] OR "Posterior colporrhaphy"[All Fields] OR "Posterior vaginal wall repair"[All Fields] 552,335

2 laparoscopic OR "laparoscopic techniques" OR laparoscopy OR "laparoscopic procedure" OR "laparoscopic surgical treatment" OR "Laparoscopy"[Mesh] OR Laparoscopies OR "Laparoscopic Surgical Procedure" OR "Laparoscopic Surgery" Most Recent "laparoscopes"[MeSH Terms] OR "laparoscopes"[All Fields] OR "laparoscope"[All Fields] OR "laparoscopical"[All Fields] OR "laparoscopically"[All Fields] OR "laparoscopics"[All Fields] OR "Laparoscopy"[MeSH Terms] OR "Laparoscopy"[All Fields] OR "laparoscopic"[All Fields] OR "laparoscopic techniques"[All Fields] OR "laparoscopie"[All Fields] OR "Laparoscopy"[MeSH Terms] OR "Laparoscopy"[All Fields] OR "laparoscopies"[All Fields] OR "laparoscopic procedure"[All Fields] OR "laparoscopic surgical treatment"[All Fields] OR "Laparoscopy"[MeSH Terms] OR "laparoscopie"[All Fields] OR "Laparoscopy"[MeSH Terms] OR "Laparoscopy"[All Fields] OR "laparoscopies"[All Fields] OR "Laparoscopic Surgical Procedure"[All Fields] OR "Laparoscopic Surgery"[All Fields] 178,113

1 "Robot-Assisted Surgery" OR Robot OR "Robotic Surgery" OR "Da Vinci" OR Aesop OR Zeus OR "Robotic Surgical Procedures"[Mesh] OR "Robot-Assisted Surgeries" OR "Robot-Assisted Surgery" OR "Robotic-Assisted Surgery" OR "Robot-Enhanced Surgery" OR "Surgery, Computer-Assisted"[Mesh] OR "Computer-Assisted Surgeries" OR "Computer-Assisted Surgery" OR "Computer Assisted Surgery" Most Recent "Robot-Assisted Surgery"[All Fields] OR "robot"[All Fields] OR "robot s"[All Fields] OR "robotically"[All Fields] OR "robotics"[MeSH Terms] OR "robotics"[All Fields] OR "robotic"[All Fields] OR "robotization"[All Fields] OR "robotized"[All Fields] OR "robots"[All Fields] OR "Robotic Surgery"[All Fields] OR "Da Vinci"[All Fields] OR "Aesop"[All Fields] OR "Zeus"[All Fields] OR "Robotic Surgical Procedures"[MeSH Terms] OR "Robot-Assisted Surgeries"[All Fields] OR "Robot-Assisted Surgery"[All Fields] OR "Robotic-Assisted Surgery"[All Fields] OR "Robot-Enhanced Surgery"[All Fields] OR "surgery, computer assisted"[MeSH Terms] OR "Computer-Assisted Surgeries"[All Fields] OR "computer-assisted surgery"[All Fields] OR "computer assisted surgery"[All Fields] 122,657

**Cochrane Library**

#1 "Robot-Assisted Surgery" OR Robot OR "Robotic Surgery" OR "Da Vinci" OR Aesop OR Zeus OR "Robot-Assisted Surgeries" OR "Robot-Assisted Surgery" OR "Robotic-Assisted Surgery" OR "Robot-Enhanced Surgery" OR "Computer-Assisted Surgeries" OR "Computer-Assisted Surgery" OR "Computer Assisted Surgery" 5282

#2 laparoscopic OR "laparoscopic techniques" OR laparoscopy OR "laparoscopic procedure" OR "laparoscopic surgical treatment" OR Laparoscopies OR "Laparoscopic Surgical Procedure" OR "Laparoscopic Surgery" 27593

#3 Burch OR Colposuspension OR Fistula OR Urethropexy OR "Pelvic organ prolapse" OR Prolapse OR Sacrohysteropexy OR Sacrocolpopexy OR Cervicosacropexy OR CSP OR "Sacral Colpopexy" OR Enterocele OR Cystocele OR Incontinence OR Incontinent OR Band OR "Type 19- Repair" OR Pectopexy OR Cervicopectopexy OR Colpopectopexy OR "Native tissue repair" OR "Anterior colporrhaphy" OR "Anterior vaginal wall repair" OR "Posterior colporrhaphy" OR "Posterior vaginal wall repair" 30224

#4 #1 AND #2 AND #3 165

**Scopus**

TITLE-ABS-KEY ( "Robot-Assisted Surgery" OR robot OR "Robotic Surgery" OR "Da Vinci" OR aesop OR zeus OR "Robot-Assisted Surgeries" OR "Robot-Assisted Surgery" OR "Robotic-Assisted Surgery" OR "Robot-Enhanced Surgery" OR "Computer-Assisted Surgeries" OR "Computer-Assisted Surgery" OR "Computer Assisted Surgery" ) AND TITLE-ABS-KEY ( laparoscopic OR "laparoscopic techniques" OR laparoscopy OR "laparoscopic procedure" OR "laparoscopic surgical treatment" OR laparoscopies OR "Laparoscopic Surgical Procedure" OR "Laparoscopic Surgery" ) AND TITLE-ABS-KEY ( burch OR colposuspension OR fistula OR urethropexy OR "Pelvic organ prolapse" OR prolapse OR sacrohysteropexy OR sacrocolpopexy OR cervicosacropexy OR csp OR "Sacral Colpopexy" OR enterocele OR cystocele OR incontinence OR incontinent OR band OR "Type 19- Repair" OR pectopexy OR cervicopectopexy OR colpopectopexy OR "Native tissue repair" OR "Anterior colporrhaphy" OR "Anterior vaginal wall repair" OR "Posterior colporrhaphy" OR "Posterior vaginal wall repair" )

2442

**Web of Science**

1: TS=("Robot-Assisted Surgery" OR Robot OR "Robotic Surgery" OR "Da Vinci" OR Aesop OR Zeus OR "Robot-Assisted Surgeries" OR "Robot-Assisted Surgery" OR "Robotic-Assisted Surgery" OR "Robot-Enhanced Surgery" OR "Computer-Assisted Surgeries" OR "Computer-Assisted Surgery" OR "Computer Assisted Surgery") Date Run: Tue Jan 30 2024 16:08:29 GMT+0300 (GMT+03:00) Results: 313028

2: TS=(laparoscopic OR "laparoscopic techniques" OR laparoscopy OR "laparoscopic procedure" OR "laparoscopic surgical treatment" OR Laparoscopies OR "Laparoscopic Surgical Procedure" OR "Laparoscopic Surgery") Date Run: Tue Jan 30 2024 16:08:45 GMT+0300 (GMT+03:00) Results: 186741

3: TS=(Burch OR Colposuspension OR Fistula OR Urethropexy OR "Pelvic organ prolapse" OR Prolapse OR Sacrohysteropexy OR Sacrocolpopexy OR Cervicosacropexy OR CSP OR "Sacral Colpopexy" OR Enterocele OR Cystocele OR Incontinence OR Incontinent OR Band OR "Type 19- Repair" OR Pectopexy OR Cervicopectopexy OR Colpopectopexy OR "Native tissue repair" OR "Anterior colporrhaphy" OR "Anterior vaginal wall repair" OR "Posterior colporrhaphy" OR "Posterior vaginal wall repair") Date Run: Tue Jan 30 2024 16:09:00 GMT+0300 (GMT+03:00) Results: 1451381

4: #3 AND #2 AND #1 Date Run: Tue Jan 30 2024 16:43:04 GMT+0300 (GMT+03:00) Results: 1059

Ovid MEDLINE(R) and Epub Ahead of Print, In-Process, In-Data-Review & Other Non-Indexed Citations, Daily and Versions <1946 to January 29, 2024>

1 Robot-Assisted Surgery.mp. or Robotic Surgical Procedures/ 18406

2 Robotics/ or Robot.mp. 47646

3 Robotic Surgery.mp. 9986

4 Da Vinci.mp. 4293

5 Aesop.mp. 234

6 Zeus.mp. 321

7 Robot-Assisted Surgeries.mp. 83

8 Robotic-Assisted Surgery.mp. 1295

9 Robot-Enhanced Surgery.mp. 5

10 Computer-Assisted Surgeries.mp. or Surgery, Computer-Assisted/ 20565

11 Computer-Assisted Surgery.mp. 1765

12 Computer Assisted Surgery.mp. 1765

13 1 or 2 or 3 or 4 or 5 or 6 or 7 or 8 or 9 or 10 or 11 or 12 79228

14 laparoscopic.mp. 136645

15 laparoscopic techniques.mp. 2253

16 Laparoscopy/ or laparoscopy.mp. 124740

17 laparoscopic procedure.mp. 2323

18 laparoscopic surgical treatment.mp. 142

19 Laparoscopies.mp. 1047

20 Laparoscopic Surgical Procedure.mp. 81

21 Laparoscopic Surgery.mp. 24068

22 14 or 15 or 16 or 17 or 18 or 19 or 20 or 21 173542

23 Burch.mp. 1318

24 Colposuspension.mp. 1075

25 Fistula.mp. or Fistula/ or Vaginal Fistula/ or Urinary Fistula/ 121159

26 Urethropexy.mp. 269

27 Pelvic organ prolapse.mp. or Pelvic Organ Prolapse/ 8633

28 Prolapse.mp. or Prolapse/ 35556

29 Sacrohysteropexy.mp. 138

30 Sacrocolpopexy.mp. 1391

31 Cervicosacropexy.mp. 17

32 CSP.mp. 6980

33 Sacral Colpopexy.mp. 330

34 Enterocele.mp. 652

35 Cystocele.mp. or Cystocele/ 1571

36 Incontinence.mp. or Urinary Incontinence/ 67877

37 Incontinent.mp. 4247

38 Band.mp. 208624

39 Type 19- Repair.mp. 0

40 Pectopexy.mp. 55

41 Cervicopectopexy.mp. 1

42 Native tissue repair.mp. 269

43 Anterior colporrhaphy.mp. 392

44 Anterior vaginal wall repair.mp. 39

45 Posterior colporrhaphy.mp. 266

46 Posterior vaginal wall repair.mp. 23

47 23 or 24 or 25 or 26 or 27 or 28 or 29 or 30 or 31 or 32 or 33 or 34 or 35 or 36 or 37 or 38 or 39 or 40 or 41 or 42 or 43 or 44 or 45 or 46 431095

48 13 and 22 and 47 1274

**Total Records: 7508**
